# Supplementary material for: A multi-center, international, randomized, 2-year, parallel-group study to assess the superiority of IVUS-guided PCI versus qualitative angio-guided PCI in unprotected left main coronary artery (ULMCA) disease: Study protocol for OPTIMAL trial
Source: PLoS One. 2022 Jan 7;17(1):e0260770. doi: 10.1371/journal.pone.0260770 (PMC8740965; doi:10.1371/journal.pone.0260770)
Supplement: S2 Appendix — (PDF) [file pone.0260770.s002.pdf]

sealed envelope™ (/)

★ THE ORIGINAL INTERNET RANDOMISATION SINCE 2001

POWER (SAMPLE SIZE) CALCULATORS

Calculate how big your clinical trial needs to be with our easy to use online calculators

There are several different sample size calculators - choose the correct one according to the type of clinical trial you are planning (superiority/equivalence/non-inferiority) and the nature of the primary outcome variable (binary/continuous).

A superiority trial is one where you want to demonstrate that one treatment or intervention is better than another (or better than no treatment/intervention). An equivalence trial is where you want to demonstrate that a new treatment is no better or worse than an existing treatment and non-inferiority is to show that a new treatment is not worse than an existing treatment.

These calculators are based on approximations to the Normal distribution and may not be suitable for small sample sizes. These calculators have been tested for accuracy (/power/validation/) against published papers.

Binary outcome superiority trial

A binary outcome has two categories, such as dead/alive, hospitalisation - yes/no, therapeutic success/failure and so on. This calculator is designed for binary outcomes in parallel group superiority trials.

The percentage of patients that meet the primary outcome definition (e.g. percentage hospitalised) is compared between two randomised groups. You should power the trial to be able to detect the smallest clinically important difference between these percentages.

Significance level (alpha)

5%

Power (1-beta)

80%

Percentage 'success' in control group

26 %

Percentage 'success' in experimental group

17 %

Calculate sample size

Sample size required per group

324

Total sample size required

648

Adjustment for non-compliance/cross-over

Percentage cross-over expected in control group

4	%

Percentage cross-over expected in experimental group

3	%

Adjusted total sample size required

750

You could say:

> 648 patients are required to have a 80% chance of detecting, as significant at the 5% level, a decrease in the primary outcome measure from 26% in the control group to 17% in the experimental group.

Technical note

Calculation based on the formula:

$$n = f(\alpha/2, \beta) \times [p_1 \times (100 - p_1) + p_2 \times (100 - p_2)] / (p_2 - p_1)^2$$

where p_1 and p_2 are the percent 'success' in the control and experimental group respectively, and

$$f(\alpha, \beta) = [\Phi^{-1}(\alpha) + \Phi^{-1}(\beta)]^2$$

Φ^{-1} is the cumulative distribution function of a standardised normal deviate.

Adjustment for cross-overs based on formula: $n_{adj} = n \times 10{,}000 / (100 - c_1 - c_2)^2$
where c_1 and c_2 are the percent cross-over in the control and experimental group respectively.

Reference

Pocock SJ. Clinical Trials: A Practical Approach. Wiley; 1983.

How to cite this service

> Sealed Envelope Ltd. 2012. Power calculator for binary outcome superiority trial. [Online] Available from: https://www.sealedenvelope.com/power/binary-superiority/ [Accessed Thu Oct 28 2021].
