# Supplementary material for: A multi-center, international, randomized, 2-year, parallel-group study to assess the superiority of IVUS-guided PCI versus qualitative angio-guided PCI in unprotected left main coronary artery (ULMCA) disease: Study protocol for OPTIMAL trial
Source: PLoS One. 2022 Jan 7;17(1):e0260770. doi: 10.1371/journal.pone.0260770 (PMC8740965; doi:10.1371/journal.pone.0260770)
Supplement: S1 Protocol — (PDF) [file pone.0260770.s003.pdf]

Clinical Study Protocol

Study title: OPtimizaTIon of Left MAin PCI with IntravascuLar Ultrasound. The OPTIMAL Randomized Controlled Trial

Short title: **OPTIMAL**

Study no.:	ECRI 13
Version no.: 2.0 (final)	Date: 3 May 2021
Sponsor:	European Cardiovascular Research Institute (ECRI) 13 B.V.
Study chairman & study principal investigator:	Prof. Adrian Banning, MD, John Radcliffe Hospital, Oxford, UK
Deputy chairman & study principal investigator:	Dr. Luca Testa, MD, PhD, IRCCS San Donato Hospital, San Donato Milanese, Milan, Italy
Country Leads:	• UK: Dr. Giovanni Luigi De Maria, MD, PhD, Oxford University Hospital, UK<br>• Italy: Dr. Francesco Bedogni, MD, San Donato, Italy<br>• Spain: Dr. José María de la Torre Hernández, MD, Santander, Spain
Contract Research Organization (CRO)	Cardialysis B.V.
Subsidizing parties:	Philips Volcano, Boston Scientific

For Publication Purposes

Provided for Publication Purposes

I Protocol Approval Page

**Study title:**	OPtimizaTIon of Left MAin PCI with IntravascuLar Ultrasound. The OPTIMAL Randomized Controlled Trial
Short title:	OPTIMAL
Study no.:	ECRI 13
Version:	2.0 (final), 3 May 2021

We, the undersigned, have read and approved the protocol specified above, and agree upon the contents:

**Study chairman & study principal investigator**<br>Prof. Adrian Banning, MD	Signature:<br><br>Date (DD-MMM-YYYY):
**Deputy chairman & study principal investigator**<br>Dr. Luca Testa, MD, PhD	Signature:<br><br>Date (DD-MMM-YYYY):
**Country Lead UK**<br>Dr. Giovanni Luigi De Maria, MD, PhD	Signature:<br><br>Date (DD-MMM-YYYY):
**Country Lead Italy**<br>Dr. Francesco Bedogni, MD, PhD	Signature:<br><br>Date (DD-MMM-YYYY):
**Country Lead Spain**<br>Dr. José María de la Torre Hernández, MD, PhD	Signature:<br><br>Date (DD-MMM-YYYY):
**ECRI representative (Sponsor)**<br>Dr. Ernest Spitzer, MD	Signature:<br><br>Date (DD-MMM-YYYY):

For Publication Purposes

II Signature Page Site Investigator

Study title:	OPtimizaTIon of Left MAin PCI with IntravascuLar Ultrasound. The OPTIMAL Randomized Controlled Trial
Short title:	OPTIMAL
Study no.:	ECRI 13
Version:	2.0 (final), 3 May 2021

I have read this protocol and/or amendment and appendices and agree to adhere to the requirements. I will provide copies of this protocol and all pertinent information to the study personnel under my supervision. I will discuss this material with them and ensure they are fully informed regarding the conduct of the study.

I agree to conduct this study according to this protocol and to comply with its requirements, ethical and safety considerations and guidelines, and to conduct the study in accordance with the ISO 14155:2020, and applicable regional regulatory requirements.

Name and title Investigator

Site name and full address

Signature Investigator

Date (DD-MMM-YYYY)

For Publication Purposes

III Protocol Outline

Study title	OPtimizaTIon of Left MAin PCI with IntravascuLar Ultrasound The OPTIMAL Randomized Controlled Trial
Short title	OPTIMAL
Study type	Randomized, controlled, multicentre, international, post-marketing strategy study
Indication	Left Main coronary artery disease treated by means of PCI
Objective	To assess the superiority of an IVUS-guided approach versus a qualitative angio-guided approach in the setting of left-main PCI.
Design	The OPTIMAL study is a randomized, controlled, multicentre, international study. A total of 800 participants will be randomized in a 1:1 fashion to IVUS-guided PCI versus qualitative angio-guided PCI, stratified by site. Patients will be consented prior to the PCI procedure and then followed up to 2 years after the index procedure.
Clinical sites	Approximately 30 sites in Europe
Investigational strategy	IVUS-guided approach in the setting of left-main PCI
Reference strategy	Qualitative angio-guided approach in the setting of left-main PCI
Inclusion criteria	1. The patient must be ≥18 years of age;<br>2. De novo lesion in an unprotected left main coronary artery (ULMCA; ostial, shaft or distal)<br>*OR*<br>ostial left anterior descending artery (LAD), or ostial circumflex artery (LCX)), both compatible with one Medina class of LM disease; or ostial intermediate branch disease.<br>3. PCI is considered appropriate and feasible by the treating interventionalist;<br>4. Silent ischemia, stable angina, unstable angina or non ST-segment elevation MI;<br>5. Able to understand and provide informed consent and comply with all study procedures, including follow-up for at least 2 years.<br>Note: A patient with a prior CABG with no patent bypass on the left main coronary artery (LMCA) can be included.

For Publication Purposes

Exclusion criteria	1. Patient is a woman who is pregnant or nursing;<br>2. Female patient of childbearing potential, i.e. who are not surgically sterile or post-menopausal (defined as no menses for 2 years without an alternative cause);<br>3. IVUS is strictly required for pre-PCI lesion severity assessment<br>4. ST-elevation myocardial infarction, cardiogenic shock;<br>5. Previous history of CABG with patent graft to the LAD and/or patent graft to the LCX;<br>6. Prior PCI of the LM, ostial LAD or ostial LCX at any time prior to enrollment;<br>7. Prior PCI of any other (i.e. non-LM, non-ostial-LAD and non-ostial-LCX) coronary artery lesions within 30 days prior to enrollment;<br>8. Patients unable to tolerate, obtain or comply with dual antiplatelet therapy for at least 6 months in stable patients and 1 year in ACS patients;<br>9. Known contraindication or hypersensitivity to everolimus, platinum-chromium, or to anticoagulants.<br>10. Patients requiring additional surgery (cardiac or non-cardiac) within 3 months post-enrollment;<br>11. Non-cardiac co-morbidities with a life expectancy less than 2 years;<br>12. Currently participating in another trial that is not yet at its primary endpoint. The patient is not allowed to participate in another investigational device or drug study for at least 12 months after enrollment.
Screening, enrollment, randomization and procedure	• Patients will be screened for inclusion/ exclusion criteria after the indication for PCI of the LM. Patients are enrolled after signing the consent form and confirmation of all inclusion / exclusion criteria, immediately followed by randomization to IVUS-guided or qualitative angiography-guided PCI with Synergy stent implantation;<br>• In the case of distal LM PCI, the bifurcation strategy is left to operator's discretion;<br>• Planned angiographic follow-up is strictly discouraged.
Non-investigational medical therapy	• Dual antiplatelet therapy must be started before the PCI;

For Publication Purposes

	• Anticoagulation during the procedure is mandatory, with type and dose left to the operator's discretion;<br>• Use of GP IIb/IIIa during the procedure is discretional.
Study endpoint(s)	Primary endpoint:<br>Patient-oriented Composite Endpoint (PoCE): all-cause death, any stroke, any myocardial infarction (MI)*, any repeat revascularization at 2 years follow-up.<br>*SCAI definition for peri-procedural MI [1]; 4th universal definition for spontaneous (>48 hours) MI [2].<br><br>Secondary endpoints:<br>1. Device-oriented Composite Endpoint (DoCE) defined as the composite of: cardiovascular death, target-vessel MI, clinically indicated repeat revascularization of the target lesion;<br>2. Vessel-oriented Composite Endpoint (VoCE) defined as the composite of: cardiovascular death, target vessel MI, repeat revascularization of the target vessel;<br>3. PoCE at 1 year<br>4. All-individual components of PoCE;<br>5. All individual components of DoCE;<br>6. All individual components of VoCE;<br>7. Definite and probable stent thrombosis according to ARCII definition;<br>8. Investigator reported hospitalization for heart failure.<br><br>Note: All endpoints will be reported at 1 and 2 years.
Study duration	Total study duration from first patient in to last patient out is expected to be approximately 4 years (2 years enrollment, 2 years follow-up).
Follow-up assessments	Clinical follow-up takes place at 30 days (telephone contact), 12 months (outpatient clinic visit or telephone contact) and 24 months (outpatient clinic visit or telephone contact) after the index procedure.
Statistical analysis	Primary analysis is performed in the modified Intention-To-Treat population. The secondary analysis is performed in the Per-protocol population (which excludes cross-overs).
Sample size	Based on previous literature[3-5], we expect an event reduction of at least 35% in the PoCE by using IVUS-guidance in Left Main

For Publication Purposes

PCI. Based on the EXCEL trial [6], the expected 2-year PoCE event rate in the IVUS guidance arm is assumed to be 17%, whereas PoCE in the angiographic guidance arm is assumed to be 26%. The cross-over rate from angio-guidance to IVUS guidance is assumed to be 4%, and the cross-over rate from IVUS guidance to angio-guidance 3%. With a two-sided type I error of 0.05, approximately 789 patients are needed to demonstrate superiority of an IVUS-guided approach versus a qualitative angio-guided approach in the setting of left-main PCI with a statistical power of 80%. A sample size of 2 x 400 patients allows for an attrition rate of 6%.

For Publication Purposes

Provided for Publication Purposes

IV Schedule of Assessments

**Event**	**Screening**	**PCI Procedure**[14]	**Post-PCI (within 24h or pre-discharge)**	**30 Days post PCI (+7 days)**	**1 Year post PCI (+ 30 days)**	**2 Years post PCI (+ 30 days)**
Type of contact	*Visit*	*Visit*	*Visit*	*Phone*	*Visit or phone*	*Visit or phone*
Informed consent	X					
Inclusion/ Exclusion criteria	X					
Vital signs[1]	X					
Medical and Cardiac History	X					
Blood laboratory[2]	X					
Cardiac biomarkers[3]	X[4]		X[5]			
12-lead ECG	X[6]		X[7]		X	X
Left Ventricular Ejection Fraction & valvular disease status[8]	X					
Cardiac medications	X	X	X	X	X	X
Anginal status	X[9]		X	X	X	X
Enrollment & randomization	X					
Angiography	X	X[10]				
IVUS[11]		X[10]				
SYNTAX Score[12]						
Serious Adverse Events reporting[13]	X	X	X	X	X	X

1. Body weight, height, blood pressure, heart rate
2. Blood laboratory (WBC, platelets, Hemoglobin, Hematocrit, and serum Creatinine) must be performed within 28 days prior to PCI procedure. HbA1c can be captured for diabetic patients.
3. Troponins (hs- I or -T), or CK-MB.
4. Local laboratory tests for cardiac biomarkers (including cardiac troponins or CK-MB, whichever used in each institution), should be done within 24 hours prior to the start of the PCI procedure, or within 72 hours for stable patients. For participants showing elevated biomarkers at baseline (i.e. above 1 ULN/URL), an additional blood sample is recommended prior to the PCI procedure (when clinically possible) to determine if biomarkers are stable, decreasing, or increasing. In stable patients, blood can be drawn from the arterial sheath prior to procedure.
5. Cardiac biomarkers are determined 6 to 24 hours after PCI procedure, or at discharge (if at least 6 hours after PCI procedure), whichever comes first. In patients with normal baseline biomarkers, if cardiac enzymes are elevated post-PCI (CK-MB > 5 ULN, or cTn/hs-cTn >35 ULN), serial measurements of cardiac enzymes must be taken until a decline is noted. In patients with elevated baseline biomarkers, if the CKMB or cTn/hs-cTn rises by an absolute increment equal to those levels recommended above, serial measurements must be taken until a decline is noted.
6. ECG acquired within 24 hours prior to PCI procedure, or within 72 hours in stable patients.
7. ECG acquired within 24 hours post-procedure.
8. LVEF & valvular disease status must be assessed within 28 days prior to enrollment, either by echocardiography, MRI, or contrast left ventriculography.
9. Prior to PCI procedure, after signing of ICF & confirmation of all in-and exclusion criteria
10. Collected centrally at the Core Lab (Cardialysis) and made available for review by the Steering Committee for feedback when required
11. Only for IVUS-arm
12. Performed by the Steering Committee (www.syntaxscore.org)
13. Starting reporting of SAEs begins directly after patient has signed informed consent, up to and including the last follow-up contact
14. Strongly recommended within 24 hours after randomization, or within 72 hours for stable patients

V Table of contents

For Publication Purposes

I PROTOCOL APPROVAL PAGE **2**
II SIGNATURE PAGE SITE INVESTIGATOR **3**
III PROTOCOL OUTLINE **4**
IV SCHEDULE OF ASSESSMENTS **8**
V TABLE OF CONTENTS **8**
VI LIST OF ABBREVIATIONS **12**
1. INTRODUCTION **13**
1.1. IVUS DEVICES 15
1.2. STENT 16
1.3. INTENDED USE UNDER INVESTIGATION 16
1.4. RISKS AND BENEFITS FOR STUDY PARTICIPANTS 16
2. STUDY OBJECTIVE **18**
2.1. STUDY HYPOTHESIS 19
3. STUDY DESIGN **19**
3.1. STUDY DESIGN SCHEMATIC 20
4. STUDY POPULATION **21**
4.1. INCLUSION CRITERIA 21
4.2. EXCLUSION CRITERIA 21
5. STUDY PROCEDURES **22**
5.1. PRE-SCREENING 22
5.2. INFORMED CONSENT & ADDITIONAL SCREENING 22
5.2.1. Informed consent in 'ad hoc' revascularization *23*
5.3. ENROLLMENT & RANDOMIZATION 23
5.4. BASELINE EVALUATION 24
5.5. LEFT MAIN TREATMENT 26
5.5.1. Angiographic eligibility criteria and general considerations for an Optimal PCI procedure *26*
5.5.2. Left Main Ostial and Shaft Lesions *28*
5.5.3. Left Main Distal Bifurcation Lesions *28*
5.5.4. Staged procedures *30*
5.5.5. Hemodynamic support *30*
5.5.6. Post-procedural IVUS *31*
5.6. OPTIMAL PCI OF OTHER CORONARY LESIONS 31
5.7. MEDICAL THERAPY 32
5.7.1. Prior to PCI procedure *32*
5.7.2. Intra-procedure adjunctive pharmacology *33*
5.7.3. Post PCI procedure *33*
5.8. BLOOD SAMPLE COLLECTION POST-PCI PROCEDURE 34
5.9. HOSPITAL DISCHARGE 35
5.10. 30 DAYS AFTER PCI (+7 DAYS): *TELEPHONE CALL* 35
5.11. YEAR 1 AFTER PCI (+ 30 DAYS) & YEAR 2 AFTER PCI (+ 30 DAYS): *ON SITE CLINICAL VISIT OR TELEPHONE CALL* 35
5.12. TRANSFER OF IVUS & ANGIO IMAGES 35
5.13. MISSED VISITS 35
5.14. LOST TO FOLLOW-UP 36
5.15. WITHDRAWALS 36
5.16. END-OF-STUDY (EOS) DEFINITION 37
6. STUDY ENDPOINTS **38**

For Publication Purposes

7. SAFETY EVALUATION AND REPORTING ... 39
7.1. SAE DEFINITIONS ... 39
7.2. REFERENCE SAFETY INFORMATION ... 39
7.3. SAE REPORTING ... 40
7.4. RISK ANALYSIS ... 40
8. STATISTICAL METHODS ... 42
8.1. ANALYSIS POPULATIONS ... 42
8.1.1. Modified intention-to-treat (mITT) population ... 42
8.1.2. Per Protocol (PP) population ... 42
8.1.3. As treated (AT) population ... 42
8.2. STATISTICAL ANALYSIS ... 42
8.2.1. Time dependent endpoints ... 42
8.3. SAMPLE SIZE DETERMINATION ... 44
9. DATA INTEGRITY AND QUALITY ASSURANCE ... 45
9.1. REGULATORY STATEMENT ... 45
9.2. DATA CAPTURING SYSTEM ... 45
9.2.1. EDC completion ... 45
9.2.2. Data recorded from screen failures ... 45
9.3. STUDY DOCUMENTATION ... 46
9.4. DATA MANAGEMENT ... 46
9.5. QUALITY ASSURANCE ... 46
9.5.1. Audit and inspection ... 46
9.5.2. Monitoring ... 47
9.6. SITE INVESTIGATOR RESPONSIBILITIES ... 47
9.7. SPONSOR RESPONSIBILITIES ... 47
9.7.1. Sponsor role ... 47
9.7.2. Delegation of sponsor tasks ... 48
9.8. ARCHIVING ... 48
9.9. END OF STUDY OR TEMPORARY HALT AND PREMATURE END OF STUDY ... 49
10. ETHICAL AND LEGAL ASPECTS ... 49
10.1. FUNDING, FINANCIAL DISCLOSURE AND INSURANCE ... 49
10.2. ETHICAL AND LEGAL CONDUCT OF THE STUDY ... 50
10.3. PROTOCOL AMENDMENTS ... 50
10.4. PATIENT INFORMATION AND CONSENT ... 50
10.5. CONFIDENTIALITY ... 50
11. PUBLICATION POLICY ... 52
12. STUDY ORGANIZATION ... 53
12.1. SPONSOR ... 53
12.2. STEERING COMMITTEE ... 53
12.3. CLINICAL EVENTS COMMITTEE ... 53
12.4. CRO ... 53
12.5. GRANT GIVERS ... 53
13. REFERENCES ... 54
14. PROTOCOL AMENDMENTS ... 57
APPENDIX: DEFINITIONS ... 58
STUDY ENDPOINTS ... 58
DEATH according to ARC-II definition [41] ... 58

For Publication Purposes

MYOCARDIAL INFARCTION....................59
CEREBROVASCULAR EVENTS (CVE) per VARC-II definition [42]....................61
REVASCULARIZATION per ARC II [41] *and EXCEL definitions* [6] 63
STENT THROMBOSIS per ARC-II definition [41]*:*....................65
Investigator-reported HOSPITALIZATION for heart failure[43] 66
OTHER DEFINITIONS....................67
ANGINA per Braunwald classification [44,45] *and Canadian Cardiovascular Society classification* [46] 67
DISSECTION, NHLBI CLASSIFICATION (National Heart Lung and Blood Institute)....................69

For Publication Purposes

VI List of abbreviations

Abbreviation	Definition	Abbreviation	Definition
(I)EC	(Independent) Ethics Committee	IRB	Institutional review board
ACT	activated clotting time	IVUS	Intravascular ultrasound
ADP	Adenosine diphosphate	LAD	Left Anterior Descending
AE	adverse event	LCx	Left Circumflex
atm	atmosphere	LIMA	Left Internal Mammary Artery
AUC	area under the curve	LM	Left Main (coronary artery)
CABG	Coronary artery bypass graft	LMCA	Left Main Coronary Artery
CI	Confidence interval	LVEF	Left Ventricular Ejection Fraction
CK-MB	creatine kinase-muscle/brain	MACE	Major adverse coronary event
CSA	cross-sectional area	MI	Myocardial infarction
cTn	Cardiac troponin	MLA	Minimum lumen area
cTn-I	Cardiac troponin type I	MLD	Minimum lumen diameter
cTn-T	Cardiac troponin type T	MRI	Magnetic resonance imaging
CTOs	Chronic total occlusions	OCT	Optical Coherence Therapy
DAPT	Dual antiplatelet therapy	OPTIMAL	OPtimizaTIon of Left MAin PCI with IntravascuLar Ultrasound. The OPTIMAL Randomized Controlled Trial
DES	Drug eluting stent	OR	Odds ratio
DFR	Diastolic hyperemia-free ratio	PCI	Percutaneous coronary intervention
DoCE	Device-oriented Composite Endpoint	POC	Polygon of Confluence
DS	Diameter stenosis	PoCE	Patient-oriented Composite Endpoint
EBC	European Bifurcation Club	POT	Proximal optimization technique
ECG	Electrocardiogram	RCA	Right coronary artery
EDC	electronic data capturing	RR	Relative risk
ePd	electronic patient dossier	SAE	Serious adverse event
FFR	fractional flow reserve	SAP	Statistical Analysis Plan
GP II b/IIIa	glycoprotein (GP) IIb/IIIa inhibitors	SCAI	Society for Cardiovascular Angiography and Interventions
cTn	high-sensitive cardiac troponin	STEMI	ST-elevation myocardial infarction
IB	Investigator's brochure	TAP	T and small protrusion
ICF	Informed Consent Form	TIMI	Thrombolysis in Myocardial Infarction
ID	ischemia-driven	ULMCA	Unprotected left main coronary artery
iFR	instant wave-free ratio	VoCE	Vessel-oriented Composite Endpoint
IFU	Instructions for use		

For Publication Purposes

1. Introduction

The atherosclerotic obstruction of the Left Main Coronary Artery (LMCA) is of particular consideration compared to other coronary segments as it usually supplies blood to >75% of the left ventricle, and untreated patients with significant disease of the LMCA have a very poor prognosis. Recent trial data have highlighted the potential of coronary stenting in the LMCA setting, particularly in those patients with less complex LMCA disease and patients unsuitable for surgery [7,8]. Angiography has suboptimal performance to assess the LMCA because of the possible lack of proximal reference and atherosclerosis within the LMCA is usually diffuse and involves the bifurcation. Calcific disease is common in the LMCA, thus a proper lesion dilation and stent expansion can be challenging. Of note, an acute complication during the treatment of LMCA can have a rapid progression towards hemodynamic instability.

In contrast to two-dimensional angiography, Intravascular Ultrasound (IVUS) is especially accurate to assess both the lumen and the features of the arterial wall and, compared to Optical Coherence Therapy (OCT) which uses infra-red light and blood clearing, has much higher tissue penetration. As such, IVUS is a particularly useful tool to assess the LMCA, considering that visual angiographic assessment, even with advanced angiographic equipment, may have a suboptimal performance [9].

The sizing of the LMCA and its bifurcation into the left anterior descending (LAD) and proximal left circumflex (LCX) arteries is predictable using fractal geometry and Murray's Law [9,10]. IVUS dimensions for a normal LMCA (or a minimally diseased LMCA) and the subtended LAD and LCX are remarkably consistent and have been confirmed in an analysis from PROSPECT (Providing Regional Observations to Study Predictors of Events in the Coronary Tree) trial [10-13].

The PROSPECT [12] trial and serial IVUS studies [14] showed that when atheroma develops, the LMCA usually undergoes positive remodeling in response to plaque deposition to preserve lumen dimensions, a process originally described by Glagov [15]. Conversely, ostial stenosis is usually a consequence of negative remodeling (sometimes without significant plaque accumulation) and can occur at all three segments – ostial LMCA, ostial LAD, and ostial LCX [16-18]. Data suggests that shorter anatomic LMCAs tend to develop ostial narrowings while longer anatomic LMCAs tend to develop distal bifurcation stenoses [19].

Careful IVUS imaging from both the LAD and the LCX back to the LMCA has demonstrated that bifurcation disease is rarely focal. In 140 patients and irrespective of angiographic Medina bifurcation classification [19], the carina and both sides of the flow divider were almost always disease-free. Continuous plaque from the LMCA into the proximal LAD artery was seen in 90% and from the LMCA into the LCX artery in 66%, with disease from the LMCA into both the LAD and LCX arteries in 62% [19]. Importantly, plaque localized to either the LAD or LCX ostium and not involving the distal LMCA was seen in only 9% of LAD arteries and 17% of LCX arteries. This typical distribution of atheroma explains why attempting to place a stent accurately at the true ostium of the LAD or LCX is usually unsuccessful and results in an angiographic result which is suboptimal - in addition to the fact that a stent in the proximal LAD can move as much as 5.5 mm between systole and diastole [19].

For Publication Purposes

Abizaid *et al* initially reported 122 patients who did not have catheter or surgical intervention related to the LMCA and who were followed for one year [20]. Logistic regression analysis identified three predictors of events: diabetes mellitus, presence of one or more major epicardial vessel or bypass graft with an >50% angiographic diameter stenosis (DS) narrowing that was not treated, and IVUS minimum lumen diameter (MLD). For the entire cohort, the event rate was 60% for an IVUS MLD < 2.0 mm, 24% for an MLD of 2.0-2.5 mm, 16% for an MLD of 2.5-3.0 mm, and 3% for an MLD >3.0 mm. Fassa *et al* reported that the mean minimum lumen area (MLA) of 121 patients with an angiographically normal or minimally diseased LMCA was 16.25 ± 4.30 mm^2 with a "lower limit of normal" (based on the mean minus two standard deviations) of 7.65 mm^2 [11]. Among a second group of 214 patients with indeterminate LMCA disease, 83 (38%) had an MLA < 7.5 mm^2, the majority of whom underwent coronary artery bypass surgery. However, there were 12/83 who did not have revascularization for various reasons including advanced age, co-morbidities, and/or previous bypass surgery and who had an extremely high rate of events. Conversely, patients with an MLA ≥ 7.5mm^2 who were treated medically or surgically or patients with an MLA < 7.5mm^2 who were treated surgically had a similar rate of events. When performing multivariate analysis, age, smoking status, and the number of diseased non-LMCA vessels remained the only significant predictors of adverse events. The LITRO Registry reported 354 patients in whom LMCA revascularization was performed in 90.5% (152/168) patients with an IVUS MLA < 6 mm^2 and was deferred in 96% (179/186) of patients with an MLA of ≥ 6mm^2 with two-year cardiac death-free survival of 97.7% in the deferred group vs 94.5% in the revascularization group (p=0.5) and an event-free survival of 87.3% vs 80.6%, respectively (p=0.3). However, there were 16 patients with an IVUS MLA of 5-6mm^2 who did not undergo revascularization because of operator and/or patient preferences and in whom cardiac death-free survival was 86% with a 62.5% survival free of cardiac death, myocardial infarction (MI), or revascularization [21].

IVUS has been compared to fractional flow reserve (FFR) in three groups of patients with inconsistent results [22-24]. Jasti *et al* initially reported that an IVUS MLA of 5.9 mm^2 (sensitivity of 93% and specificity of 95%) and an MLD of < 2.8 mm (sensitivity of 93% and specificity of 98%, respectively) for determining an FFR of < 0.75 [22]. Subsequently, Kang *et al* reported that the IVUS MLA that best predicted FFR <0.80 was < 4.8 mm^2 (89% sensitivity, 83% specificity, area under the curve [AUC] 0.90, p < 0.001) [23]. In addition, the cutoff value of plaque burden to predict FFR < 0.80 was ≥ 72% (sensitivity 73%, specificity 79%, AUC 0.79, p < 0.001). Of note, the FFR was significantly lower in 18 lesions with plaque rupture vs 37 lesions without plaque rupture (0.76 ± 0.09 vs 0.82 ± 0.09, p = 0.018). In a follow-up study done by Park *et al*, in an expanded group of 112 patients, the independent factors of an FFR of ≤ 0.80 were plaque rupture (odds ratio [OR] 4.47, p = 0.014), body mass index (OR 1.19, p = 0.05), patient age (OR 0.95, p = 0.031), and IVUS MLA (OR 0.37, p < 0.001) [24]. The optimal IVUS MLA cut-off for an FFR of ≤0.80 was 4.5 mm^2 (77% sensitivity, 82% specificity, AUC 0.83, p<0.001) with an optimal IVUS plaque burden cutoff of 77% (70% sensitivity, 82% specificity, and AUC: 0.80). The most plausible explanation for the differences between the

For Publication Purposes

minimal MLA in the studies by Jasti, Kang, and Park appeared to be the ethnicity of the patient populations as a comparison of white North American vs Asian patients showed a smaller MLA in Asian patients (5.2 ± 1.8 mm^2 vs. 6.2 ± 1.4 mm^2, p < 0.0001)[25].

Notably, given the unique prognostic implications of LM disease the European Bifurcation Club (EBC) recommended using a threshold cutoff of MLA ≤6 mm^2 to indicate an LMCA that should be treated with revascularization [26].

Recently, Ye *et al* performed a meta-analysis of 10 studies that indicated that IVUS-guided percutaneous coronary intervention (PCI) of the LMCA reduced the risk of all-cause mortality by 40% and cardiac death by 53% compared with conventional angiography-guided procedures [5]. In addition, IVUS-guided PCI was associated with lower risks of target lesion revascularization and stent thrombosis. Of the included studies, one was a small randomized trial [27]; the others were either single or multicenter registries[28-33]. The primary endpoint – all-cause mortality – was reported in nine studies; and cardiac mortality was reported in six studies. IVUS-guidance was associated with lower risks of target lesion revascularization (relative risk (RR) 0.43, 95% confidence interval (CI) 0.25–0.73, p=0.002) and definite or probable stent thrombosis (RR 0.28, 95% CI 0.12–0.67, p=0.004), but not MI or target vessel revascularization.

While most of the studies included in both meta-analyses had a significant number of distal LMCA lesions, only the propensity-score matched IVUS-TRONCO-ICP Spanish study looked at this subgroup specifically [34]. In the subgroup of distal lesions irrespective of treatment, IVUS-guidance reduce the composite of death, MI and target lesion revascularization from 19% to 11% (p = 0.03), a difference that was magnified in distal LMCA lesions treated with two stents (41.0% to 16.7%, p = 0.02). Most recently, Andell *et al* have reported a reduced incidence of a combined primary endpoint of mortality, stent thrombosis and restenosis over a period of five years when LMCA intervention was guided by IVUS [28].

Nevertheless, there is data suggesting higher volume PCI operators are likely to get better outcomes for patients with LMCA disease [29]. These single center data did not show a benefit from IVUS, and this replicates some historical data where IVUS imaging in every case was not clearly supported by data [3,30-33,35-37]. Of note, the EBC recommended IVUS in LMCA in all elective cases especially when clinical practice is evolving and when procedural complications occur [26].

For Publication Purposes

1.1. IVUS devices

The following CE-marked IVUS catheters will be used in this study:

- Opticross (HD) (manufactured by Boston Scientific Corporation, Marlborough, MA, USA) and
- Refinity- and Revolution rotational IVUS catheter, and Eagle Eye Platinum Digital IVUS catheter (manufactured by Philips Volcano, Zaventem, Belgium)

Provided for Publication Purposes

To ensure that the ratio of IVUS catheter types used is approximately 1:1 among participants randomized to the IVUS arm, one of the site selection criteria is availability of IVUS catheter type. Furthermore, the Steering Committee will monitor usage of the IVUS catheter types via the EDC. Sites that use both types will be asked to use one of the types in case of disbalance, until IVUS type usage is approximately balanced.

1.2. Stent

All LM lesions must be treated with the Synergy everolimus-eluting stents (details below) or any next generation of the Synergy stent family. Use of the Synergy stent family for *non*-LM lesions is highly recommended. The Synergy stent family has received CE-mark for the indication of this protocol and is not under investigation.

Stent name: Synergy Everolimus-Eluting Platinum Chromium Coronary Stent System (Synergy) or Synergy Megatron Everolimus-Eluting Platinum Chromium Coronary Stent System (Synergy Megatron) or any next Synergy family Stent System.

Stent manufacturer: Boston Scientific Corporation, Marlborough, MA, USA

The device has the following features:

- Backbone: Platinum-chromium
- Strut thickness: 74 µm
- Polymer coating: PLGA [Poly(D,L-lactide-co-glycolide)] bioresorbable polymer
- Polymer coating distribution: abluminal
- Polymer coating thickness: 4 µm
- Antiproliferative agent: everolimus
- Antiproliferative drug concentration: 100 µg/cm^2

Device description details of the Synergy and Synergy Megatron Stent Systems can be found in the Instructions for Use (IFU) which are provided with the product.

1.3. Intended Use Under Investigation

The IVUS catheters and stents described in section 1.1 and 1.2 are used in line with their indication.

1.4. Risks and Benefits for study participants

Coronary stenting with DES, with and without IVUS guidance, has been performed successfully for several decades. Coronary stenting with DES is considered a standard treatment for coronary revascularizations. Furthermore, there is extensive clinical and commercial experience worldwide with cardiac catheterization and interventional procedures and it is expected that the procedural risks in this study and existing stenting procedure will not be significantly different. As with any participant undergoing percutaneous coronary intervention, participants in this study may

For Publication Purposes

Provided for Publication Purposes

experience adverse events and/or outcomes. These are described in the Instructions For Use of the applicable devices used. See below for a subset of these anticipated adverse device effects, which may include, but are not necessarily limited to the following:

- Abrupt stent closure
- Allergic reaction to anti-coagulant and/or antiplatelet therapy, contrast medium, or stent materials
- Angina
- Arrhythmias, including ventricular fibrillation, ventricular tachycardia and heart block
- Arteriovenous fistula
- Cardiac arrest
- Cardiogenic shock/pulmonary edema
- Death
- Device entrapment requiring surgical intervention
- Embolization (air, tissue, or thrombotic material or material from device(s) used in the procedure); including stent embolization or migration
- Heart failure
- Hemorrhage, which may require transfusion; including (entry puncture site) bleeding and hematoma
- Hypotension/hypertension
- Infection, local or systemic; including fever and pyrogen reaction
- (Acute) myocardial ischemia or infarction
- Pain, chest or access site
- Pericardial effusion or cardiac tamponade
- Renal insufficiency or failure
- Respiratory failure
- Restenosis or aneurysm of stented segment
- Stent deformation, collapse, or fracture
- Stent & vessel thrombosis / (vessel) occlusion
- Stroke/cerebrovascular accident/transient ischemic attack
- Total occlusion of coronary artery
- Vessel trauma requiring surgical repair or reintervention; including coronary, femoral or radial artery spasm, dissection; occlusion, perforation, rupture or (pseudo)aneurysm

Known adverse events associated with daily oral administration of everolimus (or potential adverse events not captured above, that may be unique to the everolimus drug coating):

- Abdominal pain
- Acne
- Allergic/immunologic reaction to drug (everolimus or structurally related compounds) or the polymer stent coating or its individual components

For Publication Purposes

- Anemia
- Coagulopathy
- Diarrhea
- Edema
- Hemolysis
- Hypercholesterolemia
- Hyperlipidemia
- Hypertension
- Hypertriglyceridemia
- Hypogonadism male
- Leukopenia
- Liver function test abnormal
- Lymphocele
- Myalgia/Arthralgia
- Nausea
- Pain
- Pneumonia
- Pyelonephritis
- Rash
- Renal tubular necrosis
- Sepsis
- Surgical wound complication
- Thrombocytopenia
- Urinary tract infection
- Venous thromboembolism
- Viral, bacterial, and fungal infections
- Vomiting
- Wound infection

Please refer to the IFU's of the specific IVUS catheter and stent used, for their specific list of anticipated adverse device effects .

The potential benefits from this study are no different from when standard of care is followed. Possible benefits may be found for future patients treated with IVUS-guided PCI based upon results of the study.

2. Study Objective

To assess the superiority of an IVUS-guided approach versus a qualitative angio-guided approach in the setting of left-main PCI.

For Publication Purposes

2.1. Study hypothesis

In the percutaneous treatment of LM disease, an IVUS-guided strategy will be associated with less clinical events compared to an angio-guided strategy.

3. Study Design

The OPTIMAL study is a randomized, controlled, multicentre, international, post-marketing strategy study. The hypothesis is that an IVUS-guided approach is superior to a qualitative angio-guided approach in the setting of left-main PCI.

A total of 800 patients will be randomized in a 1:1 fashion to IVUS-guided PCI versus qualitative angio-guided PCI. Patients will be screened for inclusion/exclusion criteria after the indication for PCI of the LM. After confirmation of the in-and exclusion criteria and signing of informed consent form, participants are enrolled, followed by randomization to IVUS-guided or qualitative angio-guided PCI with Synergy or Synergy Megatron stent implantation. Both the participants and investigators are not blinded for the procedure. Participants are followed up for 2 years after the index procedure, with a phone call at 1 month and clinical onsite visits or phone calls at 1 and 2 years. Up to 30 sites in Europe will participate in this study.
The total study duration from first patient in to last patient out is expected to be approximately 4 years.

The OPTIMAL trial is performed in accordance with the Declaration of Helsinki, Good Clinical Practice (GCP), ISO 14155:2020, EC requirements and country specific regulations.

For Publication Purposes

Provided for Publication Purposes

3.1. Study design schematic

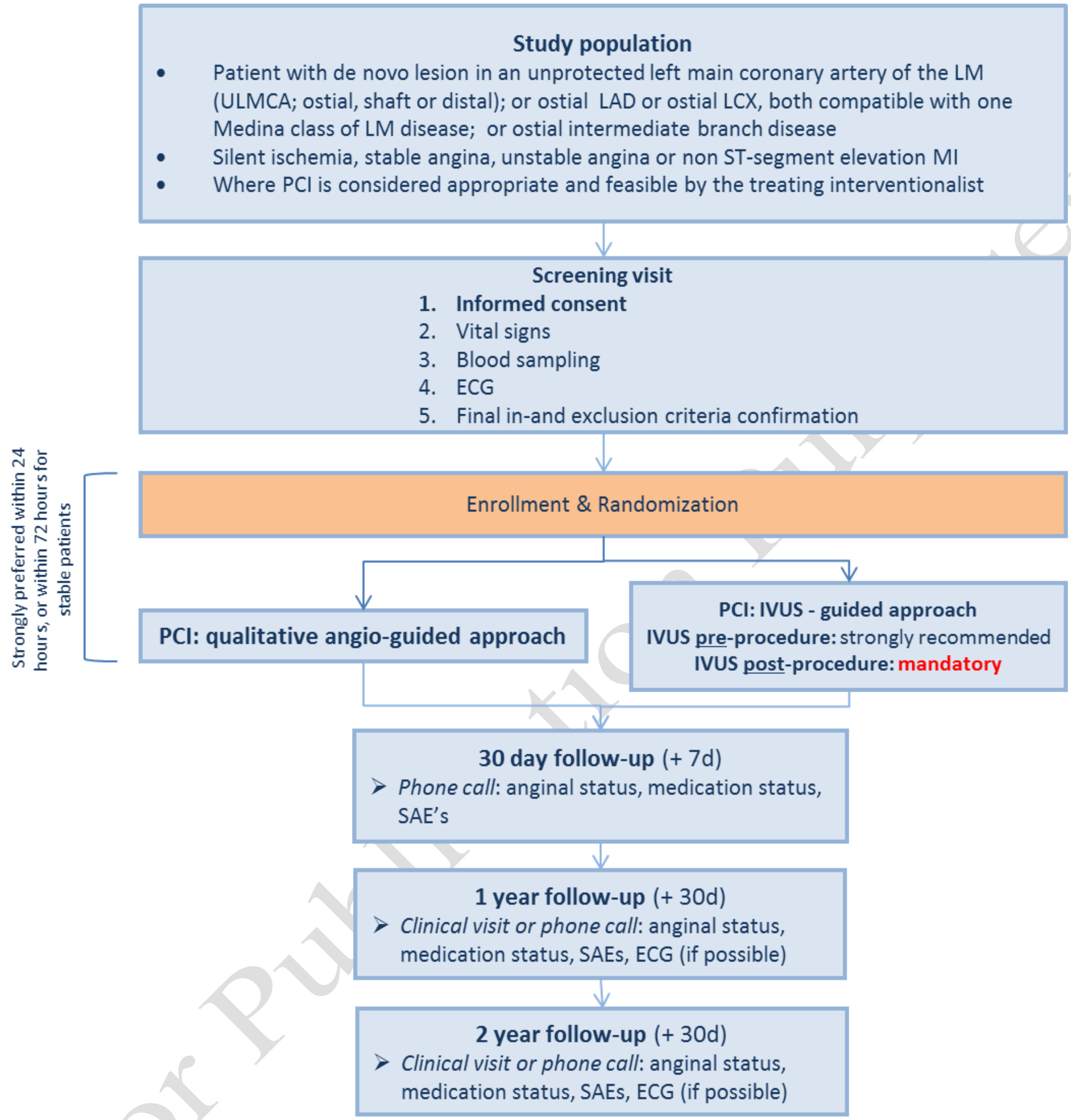

For Publication Purposes

4. Study population

This study plans to enroll 800 participants that have met all inclusion criteria and none of the exclusion criteria as delineated under section 4.1 and 1.

4.1. Inclusion Criteria

Patients to be included in the study must meet the following inclusion criteria:

1. The patient must be ≥18 years of age;
2. De novo lesion in an unprotected left main coronary artery artery (ULMCA; ostial, shaft or distal), *OR*
   ostial left anterior descending artery (LAD), or ostial circumflex artery (LCX)), both compatible with one Medina class of LM disease; or ostial intermediate branch disease;
3. PCI is considered appropriate and feasible by the treating interventionalist;
4. Silent ischemia, stable angina, unstable angina or non ST-segment elevation MI;
5. Able to understand and provide informed consent and comply with all study procedures, including follow-up for at least 2 years.

Note: A patient with a prior CABG with no patent bypass on the left main coronary artery (LMCA) can be included.

4.2. Exclusion Criteria

Patients who meet any of the following criteria will be disqualified from participation in the study:

1. Patient is a woman who is pregnant or nursing;
2. Female patient of childbearing potential, i.e. who are not surgically sterile or post-menopausal (defined as no menses for 2 years without an alternative cause);
3. IVUS is strictly required for pre-PCI lesion severity assessment
4. ST-elevation myocardial infarction, cardiogenic shock;
5. Previous history of CABG with patent graft to the LAD and/or patent graft to the LCX;
6. Prior PCI of the LM, ostial LAD or ostial LCX at any time prior to enrollment;
7. Prior PCI of any other (i.e. non-LM, non-ostial-LAD and non-ostial-LCX) coronary artery lesions within 30 days prior to enrollment;
8. Patients unable to tolerate, obtain or comply with dual antiplatelet therapy for at least 6 months in stable patients and 1 year in ACS patients;
9. Known contraindication or hypersensitivity to everolimus, platinum-chromium, or to anticoagulants.
10. Patients requiring additional surgery (cardiac or non-cardiac) within 3 months post-enrollment;
11. Non-cardiac co-morbidities with a life expectancy less than 2 years;
12. Currently participating in another trial that is not yet at its primary endpoint. The patient is not allowed to participate in another investigational device or drug study for at least 12 months after enrollment.

For Publication Purposes

5. Study procedures

A tabular schedule of assessment is provided in Section IV Schedule of Assessments.

5.1. Pre-screening

Investigators maintain a pre-screening log of all patients with a de novo lesion in the LMCA considered for enrollment, which includes no patient identifiers, but limited information like date and outcome of screening process (e.g., included in the study, or reason why not included).

5.2. Informed consent & additional screening

After Investigator or designee has screened the routinely available items and deems the patient eligible, the patient is informed about the study and asked if he / she would like to participate in the study. Protocol-specific procedures or alterations of subject care are not started until a signed informed consent is obtained. The informed consent procedure is altered in case of an 'ad hoc' revascularization (paragraph 5.2.1). Failure to obtain signed, informed consent renders the patient ineligible for the trial.

The background of the study and the benefits and risks of the procedures should be explained to the patient by the Investigator or qualified designee. Patient should be informed that (additional) screening assessments may be necessary to confirm eligibility. Patients are informed that their decision to disallow further clinical follow-up can be taken for any reason, without prejudice or detriment to their medical care, and the patient will not suffer any disadvantage as a result.

Patients must have the opportunity to ask questions and receive satisfactory answers to their inquiries, and must have adequate time to decide if they want to participate in the study.

Patients who are willing to take part in the study, will be asked to sign an ICF. For patients that are incapable of reading and / or signing, an impartial witness verifies that the information of the informed consent and any other written information was accurately explained to and understood by the patient. In such cases, the impartial witness will (co)-sign the informed consent. Additionally, the Investigator (or designee) will personally sign, and date, **and time** the form. The Investigator and/or designee must clearly document the process of obtaining informed consent in the patient's source documents. The voluntary process of obtaining informed consent confirms the patient's willingness to participate in the study. It is the Investigator's responsibility to ensure that the informed consent process is performed in accordance with the guidelines specified in section 9.1 and country specific regulations.

The patient will receive a copy of the signed informed consent for his/her records. The originally signed ICF is stored in the Investigators Site File.

The ICF will also contain the potential for possible follow-up for a total of 5 years, with data collected via telephone contacts. Follow-up up to 5 years will be performed at the sole discretion of the Sponsor and Grant givers, if funding is available.

For Publication Purposes

5.2.1. Informed consent in 'ad hoc' revascularization

In case subjects in need of 'ad hoc' revascularization, such as in NSTEMI, a staged informed consent procedure is mandatory. Subjects will be orally informed about the nature of the study by one of the investigators, witnessed at all time by independent catheterization laboratory personnel. The information will be provided after all eligibility criteria are met. The subject provides oral informed consent, and both the investigator and the witness must sign the shortened informed consent before any study procedure takes place. Directly after the procedure, and maximum within 24 hours, the subject is fully informed by means of the complete patient informed consent as described in the above paragraph. Both the patient and an investigator, or designated study personnel, must sign the complete informed consent. Both the complete and shortened signed informed consent are kept in the subject’s medical records or in the study site file, and a copy is given to the subject or the legally authorized representative. The obtaining of the consent, provisioning of a copy to the subject, along with the date **and time** are documented in the subject’s medical records. In addition, the complete and shortened signed informed consent are kept in the subject’s medical records or in the study site file and a copy is given to the subject or the legally authorized representative. Delays in obtaining the signature of the complete patient informed consent due to critical condition (e.g. patient is intubated), need to be documented in the ICF.

Consent for ‘ad hoc’ left main PCI is applicable only to centers where this approach is consistent with local practices and policies. Per-site confirmation will be documented before start of enrollment.

5.3. Enrollment & randomization

Enrollment will occur after all inclusion criteria are met and no exclusion criteria are present.
All patients participating in this clinical trial will provide informed consent prior to enrollment. Patients who were unable to provide informed consent prior to the PCI procedure cannot be enrolled.

Once patient signs ICF (or provides an oral consent with a witness, in case of ad-hoc PCIs), the Investigator or designee registers the participant in the electronic data capturing (EDC) system. The Investigator or designee is prepared to provide participant-related details, including IC date, the year of birth, age and sex. Based on this information, the system assigns a unique participant identification number.

For Publication Purposes

After enrollment and after final eligibility is established, the participant is randomized at a 1:1 ratio to IVUS-guided PCI or qualitative angio-guided PCI using the randomization module of the EDC. Stratification by site is performed to ensure balance across potential local differences in treatment practices.

If after informed consent, final eligibility cannot be established, the patient is considered a screening failure and is not randomized. All randomized patients are followed-up and included in the Full Analysis Set population.

It is strongly recommended that the PCI procedure is scheduled within 24 hours after informed consent, or within 72 hours for stable patients. If a consented patient is randomized and treated ***after*** 72 hours, reasons must be documented in the EDC. A consented patient that is not randomized within 4 weeks, will no longer be eligible and will be considered a screening failure.

5.4. Baseline evaluation

All participants will have the following assessments done prior to the PCI procedure:

- Medical and cardiac history
- Cardiac medications
- Vital signs: body weight, height, blood pressure, heart rate, within 72 hours prior to PCI
- 12-lead Electrocardiogram (ECG) acquired within 24 hours prior to PCI procedure, and within 72 hours in stable patients. The date, time, and interpretation will be recorded in the EDC.
- Left Ventricular Ejection Fraction (LVEF), assessed within 28 days prior to enrollment, either by echocardiography, magnetic resonance imaging (MRI), or contrast left ventriculography), per standard of care.
- Valvular disease status including valve type, diameter stenosis and regurgitation grade for as applicable, assessed within 28 days prior to enrollment by echocardiography, per standard of care.
- Anginal status assessed within 24 hours prior to PCI procedure
- SAEs

- **Blood sample collection prior to PCI procedure:**
    - Local laboratory tests for white blood cells, platelets, hemoglobin, hematocrit, and serum creatinine should be done *within 28 days prior to the start of PCI procedure*. HbA1c can be collected for diabetic patients.
    - Local laboratory tests for cardiac biomarkers (including cardiac troponins or CK-MB, whichever used in each institution), should be done within 24 hours prior to the start of the PCI procedure, or within 72 hours for stable patients. For participants showing elevated biomarkers at baseline (i.e. above 1 ULN/URL), an additional

For Publication Purposes

blood sample is recommended prior to the PCI procedure (when clinically possible) to determine if biomarkers are stable, decreasing, or increasing

- In stable patients, blood can be drawn from the arterial sheath prior to procedure.
- Always use the same biomarkers in every blood drawn for an individual patient; changes among different biomarkers (e.g. cTn and CK-MB) between pre-PCI and post-PCI are not allowed.
- All cardiac troponins (e.g. high-sensitivity, standard, I, T) are allowed in the study, but same tests are to be used within individual patients.
- The URL or ULN used at each center, will be captured in the EDC. If tests are performed outside the treating facility, biomarkers information should be requested to the referring center.
- Please analyze both Troponins and CK-MB when possible; in this case observations described above need to be confirmed for both biomarker types (i.e. 'and' scenarios) . If only Troponins are available or only CK-MB is available, then observations described above are to be confirmed for this unique biomarker (i.e. 'or' scenarios).

For Publication Purposes

5.5. Left Main treatment

The treatment is recommended according to the product labeling and instructions for use as well as the following recommendations:

5.5.1. Angiographic eligibility criteria and general considerations for an Optimal PCI procedure

Determination of ULMCA lesion severity:

- Unprotected left main coronary artery (ULMCA) disease with angiographic DS ≥70% (visually estimated) requiring revascularization, or:
- ULMCA disease with angiographic DS ≥ 50% but <70% (visually estimated) requiring revascularization, with one or more of the following present:
  - Non-invasive evidence of ischemia referable to a hemodynamically significant left main lesion (large area of ischemia in both the LAD and LCX territories, or in either the LAD or LCX territory in the absence of other obstructive coronary artery disease to explain the LAD or LCX defect), and/or
  - FFR ≤ 0.80 or instant wave-free ratio (iFR) ≤ 0.89, or resting full-cycle ratio (RFR) ≤ 0.89, or diastolic hyperemia-free ratio (DFR) ≤ 0.89
- If during screening (before or after consent) the patient requires use of IVUS for determination of lesion severity, the patient will disqualify for randomization (i.e. enrollment failure).
- Note: After randomization, pre-PCI IVUS use is not allowed in the angio-guided arm. If the patient nevertheless requires pre-PCI IVUS assessment, the patient becomes a cross-over and all the procedure should continue with IVUS guidance.
- In participants with "other coronary disease" outside of the ULMCA complex, the following treatment sequences are recommended:
  - If LAD and/or LCX disease is present, treat the LAD and/or LCX first (distal to proximal, as per usual PCI practice), unless the severity of the LM stenosis (e.g. > 70% stenosis) requires primary treatment of the LM first.
  - If the ULMCA lesion is critical (e.g. > 90% visually assessed stenosis or clinical instability), treat the ULMCA first, either with balloons or definitive stenting as randomized to ensure participant safety.
  - If the RCA has a severe culprit lesion in a large vessel and the ULMCA stenosis is<70%, the operator may choose to treat the RCA before the ULMCA lesion; otherwise the ULMCA lesion should usually be treated before the RCA.

For Publication Purposes

- Chronic total occlusions (CTOs) should usually be treated after completion of the ULMCA lesion (frequently as a planned second staged procedure).

- Left main lesion preparation, defined as pre-treatment with balloons or other approved devices (including rotational atherectomy, orbital atherectomy, and lithotripsy for heavily calcified vessels), of the ULMCA complex is left to the operator's best judgment, but is strongly recommended. Direct stenting of the ULMCA is strongly discouraged.

- To provide continuous branch vessel access and to ensure participant safety, two separate guidewires must always be in place in both the LAD and LCX during treatment of a distal LM bifurcation lesion.

- In all participants, it is mandatory that only the commercially available Synergy or Synergy Megatron stent systems are used. The stent systems should be used if they are available in the appropriate diameter and length (see table 1 for size matrix of the Synergy stents and table 2 for size matrix of the Synergy Megatron stents). The 4.0 mm diameter Synergy stent can be post-dilated up to 5.75 mm diameter using appropriately sized balloons. The 5.00 mm Synergy Megatron stent can be post-dilated up to 6.00 mm diameter. For additional information and sizes, please refer to the IFU.

**Table 1: Synergy stent sizes**	
Available stent lengths (mm)	8, 12, 16, 20, 24, 28, 32, 38, 48*
Available stent diameters (mm)	2.25*, 2.50, 2.75, 3.00, 3.50, 4.00

*The 48 mm length is not available in 2.25 mm diameter.

**Table 2: Synergy Megatron stent sizes**	
Available Stent Lengths (mm)	8, 12, 16, 20, 24, 28, 32
Available Stent Diameters (mm)	3.50, 4.00, 4.50, 5.00

- The following techniques are strongly recommended to optimally deploy the Synergy or Synergy Megatron DES:
  - good lesion preparation with balloons or other approved devices as above
  - selection of the stent diameter according to the distal reference vessel diameter (1.0 – 1.1: 1.0 ratio)
  - deployment of the Synergy or Synergy Megatron DES according to IFU
  - optional post-dilation using properly sized non-compliant balloons at high pressures contained within the stent margins, especially if there is an area of incomplete expansion (noted by IVUS or as a "waist" on the deployment balloon or angiographic

For Publication Purposes

narrowing post-deployment). In participants randomized to IVUS arm, post-dilatation will be performed according to IVUS guidance.
    - In the rare case where a Synergy or Synergy Megatron DES is not available in the size and/or length needed, or the Synergy or Synergy Megatron DES cannot be delivered to the target site, participants should be managed per the standard of care at the hospital site in the best interest of the participant.

- For participants allocated to IVUS arm, the criteria of IVUS guidance for ULMCA PCI are found in the IVUS acquisition protocol.

- Devices without CE Mark may not be used during the procedure.

- The provisional approach is the recommended technique for most ULMCA lesions.

5.5.2. Left Main Ostial and Shaft Lesions

- After adequate lesion preparation and in the optimal deployment view, a single stent should be implanted in the ULMCA, beginning at the ostium (placed 1-2 mm in the aorta to ensure aorto-ostial coverage) and ending in either the ULMCA segment if the ULMCA length is >8 mm, or "crossing-over" to end in either the LAD (usually) or LCX (less commonly) if the ULMCA length is < 8 mm.
- Post-dilatation with short high pressure (≥ 18 atm) non-compliant balloons within the stent margins is strongly recommended unless IVUS guidance indicates flush apposition and appropriate cross-sectional area (CSA) (minimum CSA > 8.0 mm^2).

5.5.3. Left Main Distal Bifurcation Lesions

- A single stent crossover provisional technique is strongly recommended whenever possible with the stent size selected to match the distal branch reference vessel (usually the LAD). If the side branch origin (usually the ostial LCX) has a residual stenosis <50% with Thrombolysis in Myocardial Infarction (TIMI) 3 flow, and without a significant dissection, the decision to dilate the side branch is left to the discretion of the operator.

- A careful single-stent approach with proximal optimization technique (POT) is particularly recommended because of the changes in vessel caliber proximal and distal to the LM bifurcation. Appropriate POT will reduce malapposition which is common in the LM and this may also reduce longitudinal shortening.

- If there is uncertainty concerning the adequacy of side branch result, additional physiological evaluation can be considered if the investigator deems this appropriate. It is

For Publication Purposes

strongly recommended to utilize kissing balloons after a single stent crossover technique to attempt to manage the ostial LCX without additional stent implantation. The technique of post-stent kissing balloons in this circumstance includes the use of non-compliant short balloons in both branches with balloon sizing according to the distal reference vessel diameters, initial dilation of the side branch balloon at moderate pressures (8 to 12 atm), followed by simultaneous inflation/deflation of both balloons (8 to 12 atm).

- If the side branch is still suboptimal in appearance despite multiple balloon inflations, based upon the following criteria: severe dissection (≥ grade B), TIMI flow <3, or "severe stenosis" > 70% DS (visual estimate, in the angio-guided arm) or IVUS MLA ≤4.0 mm^2 with plaque burden >60% (in the IVUS-guided arm), or FFR ≤ 0.80 – a provisional second stent should be placed.

- The technique for a provisional second stent is left to the operator's best judgment and may include any the following: T-stent, T and small protrusion (TAP), mini-crush (reverse crush), or culotte bifurcation stent techniques. The use of kissing balloons after provisional second stents is mandatory. The technique of post-stent kissing balloons in this circumstance includes the use of non-compliant short balloons in both branches with balloon sizing according to the distal reference vessel diameters, initial dilation with the side branch balloon at high pressures (≥ 18 atm), followed by simultaneous inflation and deflation of both balloons (8-12 atm).

- The decision to use a primary two stent technique strategy rather than a single crossover stent technique should be considered when the side branch (usually the LCX) is large (> 3 mm), with significant disease (by angiographic [angio-guided arm] or IVUS assessment [IVUS-guided arm]) and lesion length > 5 mm, or when there are other special anatomic considerations (e.g. heavy calcification). Considering the anatomic variability of the distal LM bifurcation, the final decision to select a primary two stent technique strategy is left to the operator's best judgment. The choice of a particular distal bifurcation stent strategy is also left to the operator's best judgment and may include T-stenting, TAP, crush, double kissing (DK)-crush or culotte stent techniques. A "V-stent" distal bifurcation stent approach and simultaneous kissing stents are strongly discouraged

- The use of kissing balloons after primary two stent technique is strongly recommended. The technique of post-stent kissing balloons in this circumstance includes the use of non-compliant short balloons within the margins of the stents in both branches with balloon sizing according to the distal reference vessel diameters, initial dilation of the side branch balloon at high pressures (≥18 atm), followed by simultaneous inflation/deflation of both balloons (8- 12 atm).

For Publication Purposes

5.5.4. Staged procedures

Given the complexity of the ULMCA participants enrolled in this clinical trial, it is anticipated that a substantial proportion of participants may fall into the category of staged procedures. In general, the decision to stage is based on patient factors (e.g. kidney function, contrast exposure, radiation exposure, patient fatigue), lesion complexity (e.g. calcifications), unexpected lengthy procedures, procedural complications or patient instability or unsuccessful first attempt.[38]

In the OPTIMAL trial, a staged procedure is defined as a planned intervention performed after the first catheterization when it fulfills the following requirements:

1) the intent to stage is documented in the EDC, provisionally or definitely, before or within 24 h after completion of the first procedure;
2) the lesion(s) to be treated during the staged procedure should be defined upfront in the EDC and should not involve the index vessel (i.e. applies only to the RCA) except if the lesions is a chronic total occlusion (e.g. CTO of LAD or LCX planned in a staged procedure);
3) the procedure must be performed within 45 days form the index PCI; and
4) stability of symptoms is required between the first and the subsequent procedure(s), because acute ischemia (including worsening of angina) would disqualify the intervention as a staged procedure.[38]

Ultimately, an independent Clinical Events Committee (CEC) determines if the second procedure is counted as a staged procedure (i.e. included in the index treatment) or as a revascularization (i.e. counted as an endpoint). Further guidance is provided in the CEC charter.
Only study stents should be used during staged procedures, if possible. During a staged procedure the same study assessments apply as during the baseline procedure. The staged procedures will not affect the original follow-up schedule.

5.5.5. Hemodynamic support

Hemodynamic support (e.g. intra-aortic balloon pump, Impella, TandemHeart) during PCI for ULMCA lesions is usually not required but there is significant variability in the perceived need for hemodynamic support among experienced operators and sites. Criteria for required hemodynamic support may include systemic hypotension, severe pulmonary hypertension, severely reduced ejection fraction, extreme anatomic complexity (e.g. severely calcified left main lesion with intended use of rotational atherectomy), and/or participant instability before or during the procedure. The decision regarding the use hemodynamic support, either elective and planned or urgently required due to participant instability, and the type of support device is left to the operator's best judgment.

For Publication Purposes

- The choice of vascular access for PCI (e.g. femoral or radial) is left to the operator's best judgment.
- The choice and use of vascular closure devices is left to the operator's best judgment.

5.5.6. Post-procedural IVUS

Post-procedural IVUS is mandatory in IVUS-guided PCI arm. All the stented LM segments (both stents in the case of 2-stent strategy) must be interrogated by IVUS before completion of the case. In Non-LM segments/vessels, IVUS post-stent optimization is highly recommended. An 8 mm^2 of final stent area must be targeted at the body of the LM, 7 mm^2 at the Polygon of Confluence (POC) of the LM; 6 mm^2 for the LAD, and 5 mm^2 for the LCX (Kang *et al* criteria[31]).
Post-PCI IVUS use is not allowed in the angio-guided arm, except if an imaging technique is strictly required for patient safety, i.e. in those cases showing an unclear angiographic result where there is a chance for a suboptimal result or a potential complication (i.e. bail-out IVUS). Refer to the IVUS acquisition protocol for details on IVUS acquisition.

5.6. Optimal PCI of Other Coronary Lesions

- Participants should be revascularized optimally based on the Investigator's discretion. This may mean the revascularization is not complete.

- For all "borderline or intermediate non-left main lesions" (40-70% diameter stenosis by angiographic visual estimate), it is strongly recommended to confirm the lesion significance before treatment using physiology (preferred, including FFR, iFR or DFR; in the angio-guided arm) or IVUS assessment (alternate; in the IVUS-guided arm). In the case of FFR, iFR or DFR this may need to be performed after successful stenting of the left main stenosis. Non-left main lesions which are not severe either by angiographic, IVUS or FFR/iFR/DFR assessment should not undergo PCI.

- For all non-left main lesions, when the participants are allocated to the IVUS guidance arm, IVUS guidance pre-treatment and assessment post-treatment to optimize lumen dimensions is recommended (especially for LAD lesions, with exceptions including distal lesions, tortuous vessels, or focal proximal lesions in large vessels).

- The liberal use of additional guidewires to protect side branches during complex angioplasty is recommended as per the operator's best judgment.

- Lesion preparation using balloons or any approved device is left to the operator's best judgment to be able to deliver the stent to the lesion and achieve full stent expansion.

For Publication Purposes

- It is mandatory that only Synergy or Synergy Megatron DES is used for all non-left main coronary lesions which are stented. If the Synergy or Synergy Megatron DES is either unavailable or cannot be delivered to lesion site, it is recommended to follow standard of care at the site.

- Liberal use of short non-compliant post-dilation balloons (≥18 atm) within the stent margins of all stents is recommended to optimize luminal results, unless IVUS otherwise shows optimal expansion and lumen dimensions.

- Criteria for optimal PCI of ULMCA and non-LM lesions are as follows: attainment of a final in-stent residual stenosis and edge stenosis of <30% as observed by QCA or <20% by visual estimation if QCA is not available.

- Specific lesion categories, such as chronic total occlusions, bifurcation disease, diffuse lesions, thrombus-containing lesions or heavily calcified lesions should be treated according to the operator's best judgment using acknowledged best PCI practices. In situations of diffuse disease or tandem lesions it is recommended to use single long stents rather than either two shorter side-by-side or overlapping stents. A liberal staging strategy should be used, especially for participants with complex double vessel or triple vessel disease, or if high doses of radiation and/or contrast are used in the first procedure

5.7. Medical therapy

5.7.1. Prior to PCI procedure

Dual antiplatelet therapy must be started before the PCI procedure.

Aspirin. A loading dose of aspirin (150–300 mg p.o. or 75–250 mg i.v.) is mandatory if the patient is not on chronic treatment with aspirin. Either regular or chewable tablets or intravenous aspirin are used for the loading dose at least 2 hours before the procedure. A loading dose in patients on chronic treatment with aspirin is not required.

Adenosine diphosphate (ADP) antagonists.

A loading dose of an ADP antagonist in ADP antagonist-naïve patients is mandatory. The choice of one of the following agents is left to the discretion of the Investigator.

- clopidogrel 600 mg before PCI; or
- at sites in countries where it is approved and is commercially available, prasugrel 60 mg at least 1 hour before PCI; or ticagrelor 180 mg at least 1 hour before PCI.

For participants already receiving chronic ADP antagonist therapy, pre-loading should follow current guidelines and local practice.

For Publication Purposes

Provided for Publication Purposes

Other medications. The use of other medication prior to PCI (e.g. statins, beta-blockers, ACE Inhibitors, unfractionated heparin, etc.) is left to the discretion of the treating physicians.

5.7.2. Intra-procedure adjunctive pharmacology

5.7.2.1. Unfractionated heparin or low molecular weight heparin

Unfractionated heparin or low molecular weight heparin is acceptable (according to proper dose guidelines, adjusted for renal insufficiency). If unfractionated heparin is used as a procedural anticoagulant, it is recommended that an initial bolus of 60 U/kg be administered, with subsequent boluses titrated to an activated clotting time (ACT) of 250 seconds. However, the exact manner of use of unfractionated heparin is left to the discretion of the operator and local practice. Low molecular weight heparin may be used as a procedural anticoagulant as per local practice and expertise, but is not recommended in participants with an estimated creatinine clearance <60 ml/min. Fondaparinux is not permitted as a procedural anticoagulant. Procedural anticoagulants should usually be discontinued at the end of the procedure, but in rare cases may be continued at low dose as per physician discretion (e.g. for participants with an indwelling intra-aortic balloon pump). The routine use of post-procedural low molecular weight heparin for prophylaxis of deep venous thrombosis is not permitted.

5.7.2.2. Glycoprotein (GP) IIb/IIIa inhibitors

GP IIb/IIIa inhibitors are strongly discouraged in participants adequately pre-loaded with an ADP antagonist (clopidogrel, prasugrel, or ticagrelor). GP IIb/IIIa inhibitors may be used, however, in participants with large amounts of thrombus, or for thrombotic or ischemic complications arising during the procedure (provisional or bail-out use) – e.g. for refractory thrombus or no reflow resistant to repeat balloon dilatation and intracoronary use of nitroprusside or calcium channel blockers in the absence of a mechanical complication. Provisional GP IIb/IIIa inhibitors may not be used for “soft” indications such as lesion haziness or a small dissection as their use in these situations will increase bleeding complications, without clear benefit.
Use of cangrelor is up to the investigator’s discretion, and should be used according to the current ESC guidelines (e.g. cangrelor may be considered in P2Y12-inhibitor naive patients undergoing PCI).

For Publication Purposes

5.7.3. Post PCI procedure

Antiplatelet therapy

Please refer to the specific package insert for clopidogrel, prasugrel or ticagrelor for indications, contraindications, warnings and precautions. Chronic daily ADP antagonist therapy is mandated for a minimum of 6 months in stable patients and 1 year in ACS patients after PCI, according to the

Provided for Publication Purposes

current ESC guidelines on myocardial revascularization. The choice of agent is left to the discretion of the Investigator, local standard of care and drug availability.

If clopidogrel is used, the maintenance dose is 75 mg/day. In case of prasugrel the maintenance dose is 10 mg/day (the dose of prasugrel may be decreased to 5mg/day in participants with a weight <60 kg or age >75 years), and in case of ticagrelor the maintenance dose is 90 mg bid.

ADP antagonists should not be discontinued after DES implantation unless absolutely necessary for major bleeding, major trauma, or major surgery necessitating discontinuation of antiplatelet therapy (e.g. intracranial surgery). Many surgeries can safely be performed while the participant is on dual antiplatelet therapy. If a participant on dual antiplatelet therapy requires surgery, strong consideration should be given to performing the surgery without antiplatelet agent discontinuation. If a particular dual antiplatelet therapy must be discontinued, a GP IIb/IIIa bridging strategy up until the time of surgery may be considered, followed by reloading of the ADP antagonist as soon as possible post-surgery.

Aspirin

Following the PCI procedure, all participants should continue on aspirin (minimum of 75 mg/day up to 162 mg/day or dose per standard hospital practice) indefinitely. Aspirin should not be discontinued for CABG or other reasons unless absolutely necessary.

Note: avoid maintenance doses of aspirin above 100 mg daily for participants prescribed with ticagrelor.

All DAPT (including start and stop times of interrupted DAPT) and other cardiac medications shall be recorded in the EDC for data collection at each visit. Extended DAPT will be at the discretion of the Investigator.

Recommendations for participants with indication for oral anticoagulation therapy (OAC)

Participants with indication for OAC should be treated as described in the current ESC guidelines, e.g. triple therapy up to 6 months (or 1 month in case of high bleeding risk), followed by dual therapy up to 12 months.

5.8. Blood sample collection post-PCI procedure

Cardiac biomarkers are determined 6 to 24 hours after PCI procedure, or at discharge (if at least 6 hours after PCI procedure), whichever comes first. In patients with normal baseline biomarkers, if cardiac enzymes are elevated post-PCI (CK-MB > 5 ULN, or cTn/hs-cTn >35 ULN), serial measurements of cardiac enzymes must be taken until a decline is noted. In patients with elevated baseline biomarkers, if the CKMB or cTn/hs-cTn rises by an absolute increment equal to those levels recommended above, serial measurements must be taken until a decline is noted.

For Publication Purposes

5.9. Hospital Discharge

At discharge, assessment of the following items will be done:

- Anginal status
- ECG: the date, time, and interpretation will be recorded in the EDC
- SAEs
- Cardiovascular medication

Furthermore, the participant must be informed of the required DAPT medication, and the follow-up visits per protocol should be scheduled.

5.10. 30 days after PCI (+7 days): *telephone call*

Assessment of:

- Anginal status
- Cardiac medication
- SAEs

5.11. Year 1 after PCI (+ 30 days) & year 2 after PCI (+ 30 days): *on site clinical visit or telephone call*

Assessment of:

- Anginal status
- 12-lead ECG (only in case of clinical visit): the date, time, and interpretation will be recorded in the EDC
- Cardiac medications
- SAEs
- Year 2 only: participant is informed that this is the end of study visit, unless decision is made to extend the study as described in section 5.16.

5.12. Transfer of IVUS & angio images

Pseudo-anonymized IVUS and angiographic images will be transferred using AG Mednet to Cardialysis, which facilitates access for image review to the Steering Committee or delegates. To ensure trial quality and adequate eligibility, every case must be submitted within 2 weeks after the PCI procedure; likewise, feedback to the sites (when required) must be provided within 2 weeks by the Steering Committee or delegates. Pseudo-anonymized images may also be sent to the grant giver(s) for research purposes after completion of the trial. The Steering Committee or delegates will provide the SYNTAX Score I into the EDC based on the images received.

5.13. Missed visits

Every effort should be made to <u>ensure participants adhere to the follow-up onsite clinical visits and phone calls. If the participant is unable to return for an onsite clinical visit or have a follow-up</u>

For Publication Purposes

Provided for Publication Purposes

phone call, the Investigator or qualified designee must document the reason the participant was unable to complete the visit, and make reasonable effort to obtain the information from the participant otherwise.

5.14. Lost to follow-up

Participants are not considered “lost to follow-up” before the end of the 2 year follow-up period. A participant is considered lost to follow-up when contact with the participant has been lost without completing the final assessment, and every attempt to contact has failed. At least 3 documented attempts must be made to contact the participant at each visit timepoint. Collection of available clinical data or vital status, either at the interventional center, at the referring hospital, with the general practitioner, or the municipal registries continues. Information regarding all attempts to contact the participant are documented and an end of study form must be completed.
Participants who are lost to follow-up will not be replaced.

5.15. Withdrawals

Study visits and data collection for all enrolled participants continue until their final visit. Participants who explicitly disallow further clinical follow-up and data collection are considered “withdrawals”. A participant’s decision to disallow further clinical follow-up can be taken for any reason, without prejudice or detriment to their medical care, and the participant will not suffer any disadvantage as a result.
If a participant disallows further collection of study data, their data are evaluated until then. Although a participant is not obliged to give his/her reasons for withdrawing prematurely from a trial, the investigator should make a reasonable effort to ascertain the reason(s), while fully respecting the participant’s rights. If available, the reason for the termination is recorded and an end of study form is completed.
Participant-specific data on the basis of material obtained before withdrawal may be generated after withdrawal (e.g. image reading, analysis of blood sample); these data will also be retained and statistically analyzed in accordance with the statistical analysis plan. The participant has the right to object to the generation and processing of this post-withdrawal data.
An excessive rate of withdrawals or lost-to-follow-up could render the study difficult to interpret. Hence, unnecessary withdrawal of participants should be avoided. Should a participant withdraw, every effort must be made to collect any data from the participant that is allowed within their consent (e.g., obtain vital status by public domain or municipality registry, even if it is obtained outside the protocol follow-up window); and observations should be completed and reported as thoroughly as possible. Obtaining vital status by public domain or municipality registry can be done even if participant withdrew the informed consent if allowed per local requirements.

Participants who have withdrawn from participation will not be replaced.

For Publication Purposes

5.16. End-of-study (EOS) definition

All participants have a final visit planned at year 2 (+ 30 days) after PCI procedure. This trial may possibly be extended with an additional follow-up via phone contact after a total of 5 years. Follow-up after 2 years and up to 5 years will be performed at the sole discretion of the Sponsor and Grant givers, if funding is available. Participants who are lost to follow-up at 2 years will be assumed as lost to follow-up permanently. The study completion is reached as soon as the final visit of the last participant is performed in all centers in all participating countries.

For Publication Purposes

6. Study endpoints

Primary endpoint:
Patient-oriented Composite Endpoint (PoCE): all-cause death, any stroke, any myocardial infarction (MI)*, any repeat revascularization at 2 years follow-up.
*SCAI definition for peri-procedural MI [1]; 4th universal definition for spontaneous (>48 hours) MI [2].

Secondary endpoints:

1. Device-oriented Composite Endpoint (DoCE) defined as the composite of: cardiovascular death, target-vessel MI, clinically indicated repeat revascularization of the target lesion;
2. Vessel-oriented Composite Endpoint (VoCE) defined as the composite of: cardiovascular death, target vessel MI, repeat revascularization of the target vessel;
3. PoCE at 1 year
4. All-individual components of PoCE;
5. All individual components of DoCE;
6. All individual components of VoCE;
7. Definite and probable stent thrombosis according to ARCII definition;
8. Investigator reported hospitalization for heart failure.

Note: All endpoints will be reported at 1 and 2 years.

For Publication Purposes

7. Safety evaluation and reporting

7.1. SAE Definitions

An AE is defined as any untoward medical occurrence, unintended disease or injury, or untoward clinical signs (including abnormal laboratory findings) in participants, users or other persons, whether or not related to the investigational medical device.

An AE is classified as “serious” if the event:

- Led to death;
- Led to serious deterioration in the health of a participant that:
  - Resulted in a life-threatening illness or injury;
  - Resulted in a permanent impairment of a body structure or a body function;
  - Required in participants hospitalization or prolongation of existing hospitalization;
  - Resulted in medical or surgical intervention to prevent permanent impairment to a body structure or a body function.
- Led to foetal distress, foetal death or a congenital abnormality or birth defect.

NOTE: Planned hospitalization for a pre-existing condition, or a procedure required by the clinical study protocol, without serious deterioration in health, is not considered a serious adverse event.

7.2. Reference Safety Information

Anticipated adverse device effects for the IVUS catheters and stents used in this study are described in the Instructions For Use, and a subset of these is described in section 1.4. For safety reporting purposes, the list of anticipated adverse device effects in section 1.4 will serve as Reference Safety Information. If the applicable device Instructions For Use’s (IFUs) are updated during the study with a significant impact, section 1.4 may be amended.

Device Malfunctions

If the Investigator observes device malfunctions that led or might have led to a death or serious deterioration in health of a participant, user or other person, or has complaints with regard to defects in the medical devices, the Investigator shall, within 24 hours of such observation, report such device malfunction or complaint to the device manufacturer, with a copy of the report to the Sponsor. The Sponsor is responsible for taking necessary actions in response to a device malfunction to protect the safety of the trial participants, e.g. temporary suspension of the trial.
If possible, the site should return the device involved in the device malfunction to the manufacturer for examination. The device manufacturer is responsible for handling all complaints and reported device malfunctions in respect of the quality of the medical device, including any measures deemed necessary, such as incident reporting to competent authorities and recalls. Discussions regarding such device malfunction or complaints will be held between the device Company and the Participating Site. Further details are described in the safety reporting plan.

For Publication Purposes

7.3. SAE Reporting

The Investigator will monitor the occurrence of SAEs for each participant during the course of the study. For the purpose of this protocol, the reporting of SAEs begins directly after participant has signed informed consent, up to and including the last follow-up contact. If an event fulfills the criteria for SAE, then this shall be reported in the EDC system without undue delay and at least within 3 calendar days of the site study staff's awareness, including the Investigators' judgment regarding causal relationship of the event to the trial procedure (if applicable) and the study device. At the time the event is reported in the EDC system no event-supporting source documentation needs to be sent. Event-supporting source documents will be requested by the sponsor for the purpose of clinical event adjudication and safety reporting. All SAEs will be followed until the event has been resolved (with or without sequelae).
Sponsor is responsible for the classification of SAEs and ongoing safety evaluation of the clinical investigation and shall review the Investigator's assessment of all SAEs and device deficiencies and determine and document in writing the sponsor's determination of seriousness and relationship to the investigational device; in case of disagreement between the sponsor and the principal Investigator(s), both opinions shall be reflected.

All devices used in this study protocol have been approved for use by the regulatory authority in the region where the study is conducted and are used within the approved indication.

Safety reporting will be in accordance with "Clinical investigation of medical devices for human patients (ISO 14155:2020) and the "guidelines on medical devices vigilance system" by the European Commission (MEDDEV2.12 rev 08, Jan 2013) and in compliance with local country law.

Primary endpoints will be collected as SAEs and presented in periodic reports, however will be excluded from expedited reporting. These include the following events:

- All-cause death
- Any Stroke
- Any Myocardial infarction (SCAI definition for peri-procedural MI (1); 4th universal definition for spontaneous (>48 hours) MI (2))
- Any Revascularization

SAE processing, distribution and reporting are detailed in the safety reporting plan.

7.4. Risk Analysis

See section 1.4 for the risk-benefit assessment, and section 9.7.1.5 for the continuous risk-benefit assessment.

For Publication Purposes

For Publication Purposes

8. Statistical methods

8.1. Analysis populations

Primary analysis will be performed on in the Modified Intention-To-Treat population. For both the Per-Protocol population and As-Treated population only the Primary endpoint, and its individual components, will be tabulated (no formal testing will be applied).

8.1.1. Modified intention-to-treat (mITT) population

The modified intention-to-treat analysis population will include all randomized participants who underwent the PCI procedure.

8.1.2. Per Protocol (PP) population

The per-protocol population will include all randomized participants in whom PCI was performed according to their randomized arm, thus excluding cross-overs, and participants of which the LM was not treated with PCI.

8.1.3. As treated (AT) population

The As Treated populated will include all randomized participants who underwent the PCI procedure, participants will be reported according to the actual treatment received.

8.2. Statistical analysis

The statistical analysis of the primary endpoint is confirmatory. The statistical analysis of the secondary endpoints is exploratory. This section indicates how the primary endpoint and the clinical secondary event points are analyzed.

8.2.1. Time dependent endpoints

Analysis of clinical primary and secondary endpoints will be performed. Two types of time-to-event, Kaplan-Meier (KM), analyses are performed. When the KM-curves for the IVUS guidance arm and the Angio Guidance arm are compared the regular KM-analysis is performed to compare the survival distributions of the two arms, using the log-rank test. This method is non-parametric.

Additionally the KM-estimates for the two arms at a single fixed time point τ (1 month, 12 months, 24 months) are compared, thus disregarding the shape of the survival curves up to time point τ. In this analysis the Com-Nougue approach will be applied.

In the Com-Nougue approach the survival proportion at time point τ is calculated for both arms.

A 95% Confidence interval and two-sided p-value will be calculated for the Risk Difference, being the difference in these survival proportions, are constructed using the Greenwood Standard Error of the survival proportions, as estimated by KM-analysis. In the calculation the complement of the survival proportions is used, measuring the incidence of the reported event.

No standard SAS procedure is available for this analysis, the analysis is therefore specified in this section.

For Publication Purposes

The Risk Difference (RD) is the difference between the two KM-estimates:

(1) $RD= KM_{Ivus\ Guidance}-KM_{Angio\ Guidance}$

A positive Risk Difference (RD>0) indicates a higher event rate for the Ivus Guidance arm than for the Angio Guidance arm, a negative Risk Difference (RD<0) indicates a lower event rate for the Ivus Guidance arm than for the Angio Guidance arm

The standard error of the Risk Difference is calculated as:
(2) $SE_{RD}=\sqrt{(SE^2_{Ivus\ Guidance}+ SE^2_{Angio\ Guidance})}$

A 95% confidence interval ($CI_{RD,95\%}$) is calculated for the risk difference by applying the standard normal distribution:

(3) $CI_{RD,95\%}=[RD-Z_{0.975}*SE_{RD} , RD+Z_{0.975}*SE_{RD}]$

where $Z_{0.975}$ is the value of the standard normal distribution which has an upper tail probability of 2.5%, this value is around +1.96.
The p-value for the superiority test is the two-sided p-value for the difference between the Risk Difference in the trial (RD) and the assumed difference under the Null Hypothesis (H_0:RD=0):

(4) $Z_{RD}= (RD-0)/SE_{RD}=RD/SE_{RD}$

The two-sided p-value (P_{RD}) for superiority is calculated applying the standard normal distribution:

(5) $P_{RD}= 2*P(Z>|Z_{RD}|)$.

When reporting the primary endpoint the IVUS guidance strategy is declared superior to the Angio Guidance strategy if the Risk Difference is negative (RD<0) and the two-sided p-value for superiority (P_{RD}) is maximum 0.05.

In case Hazard Ratios are reported the Cox proportional hazards model will be applied.

Stratified analyses according to acute coronary syndrome, sex, SYNTAX Score I, distal LM bifurcation and the presence or absence of diabetes will be carried out.

Further details of the statistical methods, analysis and handling of missing data are described in a statistical analysis plan (SAP).

For Publication Purposes

8.3. Sample Size Determination

Based on previous literature[3-5], an event reduction is assumed of at least 35% in the PoCE by using IVUS guidance in Left Main PCI. Based on data of the Excel trial, the expected 2-year PoCE in the IVUS guidance arm is assumed to be 17%, whereas PoCE in the angiographic guidance arm is assumed to be 26%.

The cross-over rate from angio-guidance to IVUS guidance is assumed to be 4%, and the cross-over rate from IVUS guidance to angio-guidance 3%. Taking these cross-over rates into account[40] and using a Z (standard normal) test for the difference between two proportion, a two-sided type I error of 0.05, approximately 789 participants are needed to demonstrate superiority of an IVUS-guided approach versus a qualitative angio-guided approach in the setting of left-main PCI with a statistical power of 80%. A sample size of 2 x 400 participants allows for an attrition rate of 6%.

For Publication Purposes

9. Data Integrity and Quality Assurance

9.1. Regulatory statement

The protocol, ICF and other study-related documents will be submitted to the (I)EC and any other regulatory body as required per local regulations. The trial will be performed in accordance with the ISO 14155:2020.

The trial will only start at a clinical site after written approval of the trial has been obtained from the appropriate national (I)EC/any other regulatory body as required per local regulations. Any additional requirements imposed by the EC or regulatory authority shall be followed, if appropriate.

9.2. Data capturing system

The data recording tool for this study will be a validated EDC system.

To protect data in the EDC system, all access to the EDC system is password-protected. All relevant study personnel (sponsor, site, Contract Research Organization (CRO) or other) seeking access to the EDC system, will follow a training before access is granted. The Investigator maintains an authorized signature log of appropriately qualified and trained site personnel to whom study duties have been delegated. All site personnel authorized to make entries and/or corrections on the EDC system are included on the authorized signature log.

All personnel with access to the EDC system is supported by a Service Desk. The EDC contains a system-generated audit trail that captures any changes made to a data field, including who made the change, why the change was made and the date and time it was made.

9.2.1. EDC completion

All data entry into the EDC system should be completed *within 5 business days* after the participant's visit/contact, or within 3 calendar days in case of SAE, to enable the monitor to review the participant's status throughout the study (see section 9.5). The participant's data in the EDC system is completed by Investigator or qualified designee, and reviewed and signed off (e-signed) by the Investigator. Data entries made in the EDC system are supported by source documents maintained for all participants enrolled in this study at the site (see section 9.3).

EDC system completion guidelines are provided to the study site staff, before the first participant is enrolled at that site.

9.2.2. Data recorded from screen failures

It may occur that a participant has signed ICF, but is considered a screen failure after additional screening. This is called a screen failure. Data of screen failures is recorded in the EDC system. At a minimum, the following data are recorded, provided that ICF has been obtained:

- Demographic information (Participant ID number; year of birth/age; sex)

For Publication Purposes

- Date of ICF
- Reason for failure of screening
- Date of last visit/contact

If a participant is deemed a screen failure, all SAEs experienced from the moment of IC signature to the moment of established screen failure are documented and reported as described in section 7.

9.3. Study Documentation

Source documents are original records containing source data. E.g., original records or certified copies of hospital records, clinical and office charts, laboratory notes, participant files, and records kept at the laboratories involved in the clinical trial). Source data could be either paper-based or electronic. It is the expectation that all data entered into the EDC has source documentation available at the site.

Per regulations described in section 9.1, the Investigator and study staff are responsible for maintaining a comprehensive and centralized file (Investigator Site File) of all study-related essential documentation, suitable for inspection at any time by representatives from sponsor and/or applicable regulatory authorities. Investigator and study staff should ensure that only authorized personnel, auditors and monitors have access to the study data.

9.4. Data management

Clinical data management will be performed in accordance with applicable CRO's standards and data cleaning procedures. This is applicable for data recorded in the EDC system as well as for data from other (external) sources (e.g. adjudication committees).

9.5. Quality assurance

Each clinical site performs internal quality management of study conduct, data and biological specimen collection, documentation and completion. Quality control procedures will be implemented beginning with the EDC and data QC checks that are run on the database. Any missing data or data anomalies will be communicated to the site(s) for clarification/resolution. In addition, monitoring visits and possibly audits and inspections ensure oversight of the full quality control process.

9.5.1. Audit and inspection

To ensure compliance with guidelines and regulations as described in section 9.1, a member of the CRO's quality assurance unit may arrange to conduct audits to assess the performance of the study at the study sites and of the study documents originating there. The Investigator will be informed of the audit outcome.

In addition, inspections by regulatory health authority representatives and (I)EC(s) are possible. The Investigator should notify the sponsor immediately of any such inspection.

Audits and inspections may occur at any time during or after completion of the study.

For Publication Purposes

9.5.2. Monitoring

In accordance with guidelines and regulations as described in 9.1, monitors contact the site prior to the start of the study to review with the site staff the protocol, study requirements, and their responsibilities to satisfy regulatory, ethical, and sponsor's requirements.

On-site monitoring visits are performed throughout the study, according to the monitoring manual, to verify adherence to the protocol/amendment(s); verify authenticity, completeness, accuracy, and consistency of the data; verify that the rights and well-being of human participants are protected; and verify adherence to guidelines and regulations as described in 9.1.

The monitor should have access to participant medical records and other study related records needed to verify the entries on the EDC. In case an electronic Patient Dossier (ePD) is used, controlled read-only access for the monitor should be arranged. If the ePD has not been validated, or the Monitor cannot be given access, a procedure must be available for generating certified copies of the source.

The monitor communicates and documents important deviations from the protocol, SOPs, guidelines and regulations as described in 9.1 to the Investigator and verifies that appropriate action designed to prevent recurrence of the detected deviations is taken. Further details are described in the monitoring manual.

9.6. Site Investigator responsibilities

Prior to starting enrollment of participants, the site Investigator must read and understand this study protocol, and sign and date the Protocol Signature page. The Investigator Site Agreement documents agreement to all conditions of the study protocol and agreement to conduct the study accordingly. The site investigator ensures that the trial is conducted according to the regulations in section 9.1.

The site Investigator, site staff and site agree to allow the monitor, auditor or inspector direct access to all relevant documents and cooperate and allocate their time to the monitor/auditor/inspector to discuss findings and any issues.

9.7. Sponsor responsibilities

9.7.1. Sponsor role

The sponsor has the overall responsibility for the conduct of the study, including assurance that the study satisfies international standards and the regulatory requirements of the relevant (competent) authorities as described in section 9.1.

9.7.1.1. General duties

Prior to allowing the sites to start enrolling participants into the study, the Sponsor is responsible for selecting Investigators, ensuring (I)EC approvals are obtained where applicable, and signing the Investigator Site Agreement with the Investigators and/or hospitals. Additionally, the Sponsor will ensure proper clinical site monitoring.

For Publication Purposes

9.7.1.2. Selection of clinical Investigators and sites

The Sponsor will select qualified Investigators and facilities which have adequate study patient population to meet the requirements of the investigation.

9.7.1.3. Training of Investigator and site personnel

The training of the Investigator and appropriate clinical site personnel will be the responsibility of the Sponsor, and may be conducted during an Investigator meeting, a site initiation visit and/or other appropriate training sessions. Before the first participant is enrolled, a training will be provided concerning the IVUS acquisition / usage (see also the IVUS acquisition protocol). Training of site staff not present during initiation visit will be responsibility of the Investigator.

9.7.1.4. Documentation

The Sponsor will collect, store, guard and ensure completion by the relevant parties of the following documents;

- All study relevant documents (e.g. protocol, (I)EC approval and comments, notification or approval and comments, patient information and informed consent template, relevant correspondence, etc.)
- Signed and dated EDC pages
- Records of any SAE reported to the Sponsor during the clinical investigation
- Any statistical analyses and underlying supporting data
- Final report and/or publication of the clinical investigation

9.7.1.5. Continuous risk-benefit analysis

The sponsor is responsible for the continuous assessment of the risk-benefit analysis throughout the study. Due to the investigator-initiated nature of this study, the Steering Committee will perform the continuous risk-benefit assessment. Details are described in the Steering Committee charter.

9.7.1.6. Other sponsor responsibilities

Other legal sponsor responsibilities including patient insurance are described in section 10.

9.7.2. Delegation of sponsor tasks

For this study, sponsor has delegated specific tasks to a Clinical Research Organization (CRO), as specified in section 12. Throughout this protocol, whenever “sponsor” is mentioned, this includes its delegate(s) as applicable.

9.8. Archiving

After completion of the study, essential documents are archived in a way that ensures that they can be made readily available upon authorities’ request. Participant's medical files should be retained in accordance with applicable legislation and in accordance with the maximum period of time permitted by site. Essential clinical trial documents (including EDC) other than participant's

For Publication Purposes

Provided for Publication Purposes

medical files must be kept for at least 15 years after completion or discontinuation of the trial. No study document should be destroyed without prior written agreement between sponsor and the Investigator. Should the Investigator wish to assign the study records to another party or move them to another location, he/she must notify sponsor in writing of the new responsible person and/or the new location.

9.9. End of study or temporary halt and premature end of study

The sponsor will notify the relevant regulatory authorities of the end of the study within a period of 90 days. The sponsor will notify the relevant regulatory authorities immediately of a temporary halt of the study, including the reason of such an action.
In case the study is ended prematurely, the sponsor will notify the relevant regulatory authorities within 15 days, including the reasons for the premature termination.
Within one year after the end of the study, the Investigator/sponsor will submit a final study report with the results of the study, or any publications/abstracts of the study, to the relevant regulatory authorities.

This study may be temporarily suspended or prematurely terminated if there is sufficient reasonable cause. Written notification, documenting the reason for study suspension or termination, will be provided by the suspending or terminating party to Investigator, funding parties and regulatory authorities. If the study is prematurely terminated or suspended, the Investigator will promptly inform the (I)EC and will provide the reason(s) for the termination or suspension. Study participants will be contacted, as applicable, and informed of changes to study visit schedule.

The study might resume once concerns are addressed, and satisfy the suspending party.

10. Ethical and legal aspects

10.1. Funding, Financial Disclosure and Insurance

This is an Investigator-initiated study under the umbrella of the European Cardiovascular Research institute (ECRI). Research grants have been provided by Boston Scientific and Philips Volcano to fund this study.

Prior to participant recruitment, the Investigator and/or Institution will sign a clinical study agreement with the Sponsor. This agreement will include the financial information including reimbursement, indemnity and insurance as agreed upon by the parties.

The sponsor maintains clinical trial insurance coverage for study participants in the event of trial-related injuries, if applicable and in accordance with the applicable laws and regulations of the

For Publication Purposes

country in which the study is performed. Also according to the applicable regulatory requirement(s), the sponsor provides insurance or indemnity (legal and financial coverage) to the Investigator/the Institution against claims arising from the trial, except for claims that arise from malpractice and/or negligence.

10.2. Ethical and Legal Conduct of the Study

The procedures set out in this protocol, pertaining to the conduct, evaluation, and documentation of this study, are designed to ensure that the sponsor and Investigator adhere to the guidelines as specified in section 9.1.

Strict adherence to all specifications laid down in this protocol is required for all aspects of study conduct; the Investigator may not modify or alter the procedures described in this protocol.

Modifications to the study protocol will not be implemented by either the sponsor or the Investigator without agreement by both parties. However, the Investigator or the sponsor may implement a deviation from, or a change of, the protocol to eliminate any immediate hazard(s) to the trial participants without prior (I)EC/sponsor approval/favorable opinion. As soon as possible, the implemented deviation or change, the reasons for it and if appropriate the proposed protocol amendment should be submitted to the (I)EC/head of medical institution/sponsor.

10.3. Protocol amendments

Any amendments to the study protocol that seem to be appropriate as the study progresses will be communicated to the Investigator by the Sponsor. All substantial protocol amendments will undergo the same review and approval process as the original protocol.

All substantial and non-substantial amendments are handled according to local regulations, and may be submitted for notification or approval to local authorities. Changes may be implemented after the protocol amendment has been approved by the IEC, unless immediate implementation of the change is necessary for participant safety.

10.4. Patient Information and Consent

The ICF and any other written information provided to participants will be revised whenever important new information becomes available that may be relevant to the participant’s consent, or there is an amendment to the protocol that requires a change to the content of the participant information and / or the written ICF. The Investigator will inform the participant of changes in a timely manner and will ask the participant to confirm his/her participation in the study by signing the revised ICF. Any revised written ICF and written information must receive the (I)EC`s approval / favorable opinion in advance of use.

10.5. Confidentiality

All records identifying the participant will be kept confidential and, to the extent permitted by the applicable laws and/or regulations, will not be made publicly available. Participant names will not be supplied to the sponsor. Only the participant number will be recorded in the EDC, if the participant name appears on any other document (e.g. angiogram, IVUS, ECG), it must be

For Publication Purposes

obliterated before a copy of the document is supplied to the sponsor. Images sent to the Principal Investigators for feedback are pseudo-anonymized, removing all patient identifiers except the date of birth. Study findings stored on a computer will be stored in accordance with local data protection laws. If the results of the study are published, the participant's identity will remain confidential. The Investigator will maintain a list to enable participants to be identified. As long as it is necessary to be able to trace data to an individual participant (15 years), a participant identification code list can be used to link the data to the participant. The code should not be based on the patient initials and birth-date. The key to the code should be safeguarded by the Investigator. The handling of personal data will comply with the EU General Data Protection Regulation and national regulations.

For Publication Purposes

Provided for Publication Purposes

11. Publication policy

The Steering Committee and Investigators are committed to the publication and widespread dissemination of the results of the study. Data from this study will not be withheld regardless of the findings.

The OPTIMAL study is an Investigator-initiated and scientifically driven study nested within ECRI and set up in collaboration with Boston Scientific and Philips Volcano. All public presentations and manuscript generation and submissions will be led under the auspices of the Steering Committee. However, this study represents a joint effort between Investigators, ECRI and collaborators, and as such, the parties agree that the recommendation of any party concerning manuscripts or text shall be taken into consideration in the preparation of final scientific documents for publication or presentation.

The final locked database will be housed at the data management center at Cardialysis. Cardialysis will not publicly release data or study-related material, presentations, or manuscripts without the express permission of the Steering Committee. Before publication of the main results, the PIs have full access to the data (i.e. all requested results are made available). After the main publication of the primary endpoint, PIs, high enrollers, and the manufacturers may request sub-analyses under the auspices of the Steering Committee. Proposals are submitted to the Sponsor which liaises with the Steering Committee to determine priority, relevance, scientific value and novelty. Approved sub-analyses are charged to the statistical department of Cardialysis. The statistical analysis database may be distributed to the members of the Steering Committee within the Sponsor's publication policy for sub-sequent sub-analyses. The publication and/or presentation of results from a single trial site are not allowed until publication and/or presentation of the multi-center results. All single site data for public dissemination must be generated from the central database – local database projects are not permitted. All proposed publications and presentations resulting from or relating to the study (whether from multicenter data or single site analysis) must be submitted to the Steering Committee and the Sponsor for review and approval prior to submission for publication or presentation.

For Publication Purposes

12. Study Organization

12.1. Sponsor

ECRI-13 b.v.
Westblaak 98
3012 KM
Rotterdam

12.2. Steering Committee

The Steering Committee is responsible for the overall design, conduct, and supervision of the study, including the development of any protocol amendments. The Steering Committee also reviews the progress of the study at regular intervals to ensure participant safety and study integrity. The Steering Committee will allocate appropriately trained personnel to perform monitoring on the study. The composition of the Steering Committee and additional details are described in the Steering Committee charter.

12.3. Clinical Events Committee

Clinical events are independently reviewed and adjudicated in a blinded manner by a Clinical Events Committee. Refer to the appendix for details on the definitions of clinical events. Further details on endpoint sub-categories and other CEC processes are provided in the CEC charter.

12.4. CRO

Cardialysis B.V.
Westblaak 98
3012 KM, Rotterdam
The Netherlands

12.5. Grant givers

Boston Scientific
300 Boston Scientific Way
Marlborough, MA 01752-1234
United States

Philips Volcano
Excelsiolaan 41,
1930 Zaventem, Belgium

For Publication Purposes

13. References

1. Moussa ID, Klein LW, Shah B, et al. Consideration of a new definition of clinically relevant myocardial infarction after coronary revascularization: an expert consensus document from the Society for Cardiovascular Angiography and Interventions (SCAI). *Catheterization and cardiovascular interventions : official journal of the Society for Cardiac Angiography & Interventions* 2014; **83**(1): 27-36.

2. Thygesen K, Alpert JS, Jaffe AS, et al. Fourth universal definition of myocardial infarction (2018). *European heart journal* 2018.

3. Park SJ, Kim YH, Park DW, et al. Impact of intravascular ultrasound guidance on long-term mortality in stenting for unprotected left main coronary artery stenosis. *Circulation Cardiovascular interventions* 2009; **2**(3): 167-77.

4. Testa L, Latib A, Bollati M, et al. Unprotected left main revascularization: Percutaneous coronary intervention versus coronary artery bypass. An updated systematic review and meta-analysis of randomised controlled trials. *PloS one* 2017; **12**(6): e0179060.

5. Ye Y, Yang M, Zhang S, Zeng Y. Percutaneous coronary intervention in left main coronary artery disease with or without intravascular ultrasound: A meta-analysis. *PloS one* 2017; **12**(6): e0179756.

6. Stone GW, Kappetein AP, Sabik JF, et al. Five-Year Outcomes after PCI or CABG for Left Main Coronary Disease. *The New England journal of medicine* 2019; **381**(19): 1820-30.

7. Makikallio T, Holm NR, Lindsay M, et al. Percutaneous coronary angioplasty versus coronary artery bypass grafting in treatment of unprotected left main stenosis (NOBLE): a prospective, randomised, open-label, non-inferiority trial. *Lancet (London, England)* 2016; **388**(10061): 2743-52.

8. Stone GW, Sabik JF, Serruys PW, et al. Everolimus-Eluting Stents or Bypass Surgery for Left Main Coronary Artery Disease. *The New England journal of medicine* 2016; **375**(23): 2223-35.

9. Motreff P, Rioufol G, Gilard M, et al. Diffuse atherosclerotic left main coronary artery disease unmasked by fractal geometric law applied to quantitative coronary angiography: an angiographic and intravascular ultrasound study. *EuroIntervention : journal of EuroPCR in collaboration with the Working Group on Interventional Cardiology of the European Society of Cardiology* 2010; **5**(6): 709-15.

10. Finet G, Gilard M, Perrenot B, et al. Fractal geometry of arterial coronary bifurcations: a quantitative coronary angiography and intravascular ultrasound analysis. *EuroIntervention : journal of EuroPCR in collaboration with the Working Group on Interventional Cardiology of the European Society of Cardiology* 2008; **3**(4): 490-8.

11. Fassa AA, Wagatsuma K, Higano ST, et al. Intravascular ultrasound-guided treatment for angiographically indeterminate left main coronary artery disease: a long-term follow-up study. *Journal of the American College of Cardiology* 2005; **45**(2): 204-11.

For Publication Purposes

12. Inaba S, Mintz GS, Shimizu T, et al. Compensatory enlargement of the left main coronary artery: insights from the PROSPECT study. *Coronary artery disease* 2014; **25**(2): 98-103.

13. Kim SG, Apple S, Mintz GS, et al. The importance of gender on coronary artery size: in-vivo assessment by intravascular ultrasound. *Clinical cardiology* 2004; **27**(5): 291-4.

14. Von Birgelen C, Hartmann M, Mintz GS, et al. Spectrum of remodeling behavior observed with serial long-term (>/=12 months) follow-up intravascular ultrasound studies in left main coronary arteries. *The American journal of cardiology* 2004; **93**(9): 1107-13.

15. Glagov S WE, Zarins CK, Stankunavicius R, Kolettis GJ. Compensatory enlargement of human atherosclerotic coronary arteries. *The New England journal of medicine* 1987; **317**(25): 1604.

16. Fujii K, Kobayashi Y, Mintz GS, et al. Dominant contribution of negative remodeling to development of significant coronary bifurcation narrowing. *The American journal of cardiology* 2003; **92**(1): 59-61.

17. Kim SW, Mintz GS, Ohlmann P, et al. Comparative intravascular ultrasound analysis of ostial disease in the left main versus the right coronary artery. *The Journal of invasive cardiology* 2007; **19**(9): 377-80.

18. Maehara A, Mintz GS, Castagna MT, et al. Intravascular ultrasound assessment of the stenoses location and morphology in the left main coronary artery in relation to anatomic left main length. *The American journal of cardiology* 2001; **88**(1): 1-4.

19. Oviedo C, Maehara A, Mintz GS, et al. Intravascular ultrasound classification of plaque distribution in left main coronary artery bifurcations: where is the plaque really located? *Circulation Cardiovascular interventions* 2010; **3**(2): 105-12.

20. Abizaid AS, Mintz GS, Abizaid A, et al. One-year follow-up after intravascular ultrasound assessment of moderate left main coronary artery disease in patients with ambiguous angiograms. *Journal of the American College of Cardiology* 1999; **34**(3): 707-15.

21. de la Torre Hernandez JM. State-of-the-Art Review: Intravascular Imaging and Physiology in the Assessment of Non-LMCA and LMCA Stenosis Severity. 2017.

22. Jasti V, Ivan E, Yalamanchili V, Wongpraparut N, Leesar MA. Correlations between fractional flow reserve and intravascular ultrasound in patients with an ambiguous left main coronary artery stenosis. *Circulation* 2004; **110**(18): 2831-6.

23. Kang SJ, Lee JY, Ahn JM, et al. Intravascular ultrasound-derived predictors for fractional flow reserve in intermediate left main disease. *JACC Cardiovascular interventions* 2011; **4**(11): 1168-74.

24. Park SJ, Ahn JM, Kang SJ, et al. Intravascular ultrasound-derived minimal lumen area criteria for functionally significant left main coronary artery stenosis. *JACC Cardiovascular interventions* 2014; **7**(8): 868-74.

For Publication Purposes

25. Rusinova RP, Mintz GS, Choi SY, et al. Intravascular ultrasound comparison of left main coronary artery disease between white and Asian patients. *The American journal of cardiology* 2013; **111**(7): 979-84.

26. Mintz GS, Lefevre T, Lassen JF, et al. Intravascular ultrasound in the evaluation and treatment of left main coronary artery disease: a consensus statement from the European Bifurcation Club. *EuroIntervention : journal of EuroPCR in collaboration with the Working Group on Interventional Cardiology of the European Society of Cardiology* 2018; **14**(4): e467-e74.

27. Tan Q, Wang Q, Liu D, Zhang S, Zhang Y, Li Y. Intravascular ultrasound-guided unprotected left main coronary artery stenting in the elderly. *Saudi medical journal* 2015; **36**(5): 549-53.

28. Andell P, Karlsson S, Mohammad MA, et al. Intravascular Ultrasound Guidance Is Associated With Better Outcome in Patients Undergoing Unprotected Left Main Coronary Artery Stenting Compared With Angiography Guidance Alone. *Circulation Cardiovascular interventions* 2017; **10**(5).

29. Gao XF, Kan J, Zhang YJ, et al. Comparison of one-year clinical outcomes between intravascular ultrasound-guided versus angiography-guided implantation of drug-eluting stents for left main lesions: a single-center analysis of a 1,016-patient cohort. *Patient preference and adherence* 2014; **8**: 1299-309.

30. Jama A CF, Lennon R, Lerman A. The clinical impact of intravascular ultrasound in patients undergoing implantation of drug-eluting stents in the left main. *J Am Coll Caardiol* 2011; **58 (20)**: B167.

31. Kang SJ, Ahn JM, Song H, et al. Comprehensive intravascular ultrasound assessment of stent area and its impact on restenosis and adverse cardiac events in 403 patients with unprotected left main disease. *Circulation Cardiovascular interventions* 2011; **4**(6): 562-9.

32. Kinoshita N, Ohota K, Yamada T, et al. AS-138: Clinical Long-Term Outcomes after DES Stenting with or without Intravascular Ultrasound Guidance. *American Journal of Cardiology* 2010; **105**(9): 59A.

33. Narbute I KI, Trusinskis K, Sondore D, Jegere S, Latkovskis G, et al. Better one-year survival in consecutive unprotected left main patients with cutting balloon pre-dilatation and IVUS guidance. *EuroIntervention : journal of EuroPCR in collaboration with the Working Group on Interventional Cardiology of the European Society of Cardiology* 2012; **8**.

34. de la Torre Hernandez JM, Baz Alonso JA, Gomez Hospital JA, et al. Clinical impact of intravascular ultrasound guidance in drug-eluting stent implantation for unprotected left main coronary disease: pooled analysis at the patient-level of 4 registries. *JACC Cardiovascular interventions* 2014; **7**(3): 244-54.

35. Park SH RS, Cho AR, Lee HG, Lee SW, Shin WY, et al. . Impact of intravascular ultrasound guided left main intervention with drug-eluting stents on 2-year clinical outcomes. *The American journal of cardiology* 2012; **109 (7)**.

For Publication Purposes

36. Tang Y, Tian, J., Guan, C., Wang, W., Zhang, K., Chen, J., Wu, Y.,Yan, H., Zhao, Y., Qiao, S., Yang, Y., Mintz, G., Xu, B., . TCT-555 Intravascular Ultrasound Guidance Improves the Long-term Prognosis in Patients with Unprotected Left Main Coronary Artery Disease Undergoing Percutaneous Coronary Intervention. *JACC* 2016; **68**(18): B224.

37. Tian J, Guan C, Wang W, et al. Intravascular Ultrasound Guidance Improves the Long-term Prognosis in Patients with Unprotected Left Main Coronary Artery Disease Undergoing Percutaneous Coronary Intervention. *Scientific reports* 2017; **7**(1): 2377.

38. Spitzer E, McFadden E, Vranckx P, et al. Defining Staged Procedures for Percutaneous Coronary Intervention Trials: A Guidance Document. *JACC Cardiovascular interventions* 2018; **11**(9): 823-32.

39. Neumann FJ, Sousa-Uva M, Ahlsson A, et al. 2018 ESC/EACTS Guidelines on myocardial revascularization. *European heart journal* 2019; **40**(2): 87-165.

40. Pocock SJ. Clinical Trials - a practical approach: John Wiley & Sons; 1983.

41. Garcia-Garcia HM, McFadden EP, Farb A, et al. Standardized End Point Definitions for Coronary Intervention Trials: The Academic Research Consortium-2 Consensus Document. *Circulation* 2018; **137**(24): 2635-50.

42. Kappetein AP, Head SJ, Genereux P, et al. Updated standardized endpoint definitions for transcatheter aortic valve implantation: the Valve Academic Research Consortium-2 consensus document. *Journal of the American College of Cardiology* 2012; **60**(15): 1438-54.

43. Hicks KA, Tcheng JE, Bozkurt B, et al. 2014 ACC/AHA Key Data Elements and Definitions for Cardiovascular Endpoint Events in Clinical Trials: A Report of the American College of Cardiology/American Heart Association Task Force on Clinical Data Standards (Writing Committee to Develop Cardiovascular Endpoints Data Standards). *Journal of the American College of Cardiology* 2015; **66**(4): 403-69.

44. Hamm CW, Braunwald E. A classification of unstable angina revisited. *Circulation* 2000; **102**(1): 118-22.

45. Braunwald E. Unstable angina. A classification. *Circulation* 1989; **80**(2): 410-4.

46. Campeau L. Letter: Grading of angina pectoris. *Circulation* 1976; **54**(3): 522-3.

14. Protocol amendments

Not applicable

For Publication Purposes

Appendix: Definitions

STUDY ENDPOINTS

DEATH according to ARC-II definition [41]

**Type of Death**	**Definition**
Cardiovascular death*	Cardiovascular death is defined as death resulting from cardiovascular causes. The following categories may be collected:<br>1. Death caused by acute MI<br>2. Death caused by sudden cardiac, including unwitnessed, death<br>3. Death resulting from heart failure<br>4. Death caused by stroke<br>5. Death caused by cardiovascular procedures<br>6. Death resulting from cardiovascular hemorrhage<br>7. Death resulting from other cardiovascular cause
Noncardiovascular death	Noncardiovascular death is defined as any death that is not thought to be the result of a cardiovascular cause. The following categories may be collected:<br>1. Death resulting from malignancy<br>2. Death resulting from pulmonary causes<br>3. Death caused by infection (includes sepsis)<br>4. Death resulting from gastrointestinal causes<br>5. Death resulting from accident/trauma<br>6. Death caused by other noncardiovascular organ failure<br>7. Death resulting from other noncardiovascular cause
Undetermined	Undetermined cause of death is defined as a death not attributable to any other category because of the absence of any relevant source documents. Such deaths are classified as cardiovascular for end point determination.**

* Cardiovascular death will be classified as cardiac or vascular, as described in the CEC charter.
** Undetermined deaths are classified as cardiac, and thus, cardiovascular.

For Publication Purposes

MYOCARDIAL INFARCTION

4th universal definition for spontaneous (>48 hours) MI [2]. SCAI definition for peri-procedural MI[1].

Fourth Universal definition of myocardial injury and myocardial infarction
**Criteria for myocardial injury**
The term myocardial injury should be used when there is evidence of elevated cardiac troponin values (cTn) with at least one value above the 99th percentile upper reference limit (URL). The myocardial injury is considered acute if there is a rise and/or fall of cTn values.
**Criteria for acute myocardial infarction (types 1, 2 and 3 MI)**
The term acute myocardial infarction should be used when there is acute myocardial injury with clinical evidence of acute myocardial ischaemia and with detection of a rise and/or fall of cTn values with at least one value above the 99th percentile URL and at least one of the following:<br>• Symptoms of myocardial ischaemia;<br>• New ischaemic ECG changes;<br>• Development of pathological Q waves;<br>• Imaging evidence of new loss of viable myocardium or new regional wall motion abnormality in a pattern consistent with an ischaemic aetiology;<br>• Identification of a coronary thrombus by angiography or autopsy (not for types 2 or 3 MIs).<br>Post-mortem demonstration of acute athero-thrombosis in the artery supplying the infarcted myocardium meets criteria for *type 1 MI*.<br>Evidence of an imbalance between myocardial oxygen supply and demand unrelated to acute athero-thrombosis meets criteria for *type 2 MI*.<br>Cardiac death in patients with symptoms suggestive of myocardial ischaemia and presumed new ischaemic ECG changes before cTn values become available or abnormal meets criteria for *type 3 MI*.
**Criteria for coronary procedure-related myocardial infarction (types 4 and 5 MI) -**
The SCAI definition will be used.
**Criteria for prior or silent/unrecognized myocardial infarction**
Any one of the following criteria meets the diagnosis for prior or silent/unrecognized MI:<br>• Abnormal Q waves with or without symptoms in the absence of non-ischaemic causes.<br>• Imaging evidence of loss of viable myocardium in a pattern consistent with ischaemic aetiology.<br>• Patho-anatomical findings of a prior MI.

Peri-procedural MI according to SCAI 2013 definition
**Peri-procedural MI after PCI or CABG (<48 hours post- PCI or CABG)**
For patients with normal baseline cardiac biomarkers: any of the following criteria:<br>• CK-MB ≥10×ULN or cTn (I or T) ≥70×ULN<br>• OR: CK-MB ≥ 5×ULN or cTn (I or T) ≥35×ULN in combination with any of the following:<br>- New pathologic Q-waves in ≥2 contiguous leads<br>- OR: new persistent LBBB<br><br>*For patients with elevated baseline cardiac biomarkers: any of the following criteria:*<br>• *When biomarker levels are stable or falling*, there should be new CK-MB elevation by an absolute increment of ≥10×ULN

Peri-procedural MI according to SCAI 2013 definition
(or ≥70×ULN for cTnI or T) from the previous nadir level<br>• *When biomarker levels have not been shown to be stable or falling*, there should be a further rise in CK-MB or troponin beyond the most recently measured value by an absolute increment of ≥10×ULN in CK-MB or ≥70×ULN in cTn plus new ST-segment elevation or depression plus signs consistent with a clinically relevant MI, such as new onset or worsening heart failure or sustained hypotension.<br><br>While not currently recommended as part of this definition, use of post-CABG ECGs, indices of hemodynamic instability, and imaging studies demonstrating new wall motion abnormalities are suggested to complement biomarker elevations post- CABG to improve specificity.

In addition, the following electrocardiographic classification of myocardial infarction will be applied:

- Q-wave MI
- Non Q-wave MI

Finally, the relationship of each myocardial infarction with the target vessel will be adjudicated by the CEC. Any myocardial infarction not clearly attributable to a non-target vessel will be considered related to the target vessel.

Target Vessel Myocardial Infarction

Myocardial Infarction not clearly attributable to a non-target vessel.

For Publication Purposes

CEREBROVASCULAR EVENTS (CVE) per VARC-II definition [42]

Diagnostic criteria

Acute episode of a focal or global neurological deficit with at least one of the following: change in the level of consciousness, hemiplegia, hemiparesis, numbness, or sensory loss affecting one side of the body, dysphasia or aphasia, hemianopia, amaurosis fugax, or other neurological signs or symptoms consistent with stroke.

Stroke: duration of a focal or global neurological deficit >24 h; OR <24 h if available neuroimaging documents a new haemorrhage or infarct; OR the neurological deficit results in death.

TIA: duration of a focal or global neurological deficit <24 h, any variable neuroimaging does not demonstrate a new hemorrhage or infarct. No other readily identifiable non-stroke cause for the clinical presentation (e.g. brain tumour, trauma, infection, hypoglycemia, peripheral lesion, pharmacological influences), to be determined by or in conjunction with the designated neurologist.*

Confirmation of the diagnosis by at least one of the following: Neurologist or neurosurgical specialist Neuroimaging procedure (CT scan or brain MRI), but stroke may be diagnosed on clinical grounds alone.

Stroke classification

Ischemic: an acute episode of focal cerebral, spinal, or retinal dysfunction caused by infarction of the central nervous system tissue.

Hemorrhagic: an acute episode of focal or global cerebral or spinal dysfunction caused by intraparenchymal, intraventricular, or subarachnoid hemorrhage.

A stroke may be classified as **undetermined** if there is insufficient information to allow categorization as ischemic or haemorrhagic.

Stroke definitions†

Disabling stroke: a modified Rankin score (mRs) of 2 or more at 90 days and an increase in at least one mRS category from an individual's pre-stroke baseline.

Non-disabling stroke: an mRS score of 2 at 90 days or one that does not result in an increase in at least one mRS category from an individual's pre-stroke baseline.

**Patients with non-focal global encephalopathy will not be reported as a stroke without unequivocal evidence of cerebral infarction-based upon neuroimaging studies (CT scan or Brain MRI).*
†Modified Rankin Scale assessments should be made by qualified individuals.

Stroke disability

For Publication Purposes

Stroke disability will be classified using an adaptation of the modified Rankin Scale as follows, the assessment of which will be based on the Modified Rankin Disability Questionnaire:

Scale	Disability
0	No stroke symptoms at all. (May have other complaints)
1	No significant disability despite persistent stroke symptoms. Able to carry out all usual duties and activities.
2	Slight disability. Unable to carry out usual activities, but able to look after affairs without assistance. Could live alone.
3	Moderate disability. Requiring some help, but able to walk without assist (of a person). Can be left alone for a few days.
4	Moderate to severe disability. Unable to walk without assist (of a person). Unable to attend to own bodily needs without assist. Could be left alone for a few hours of a day.
5	Severe disability. Bedridden, incontinent, and requiring constant nursing care and attention and 24 hour supervision.
6	Dead.

For Publication Purposes

REVASCULARIZATION per ARC II [41] and EXCEL definitions [6]

A coronary revascularization procedure may be either a coronary artery bypass graft (CABG) surgery or a percutaneous coronary intervention (PCI) (for example, angioplasty, stenting), regardless of procedural success.

Coronary revascularization procedures may be further classified as follows:

- Urgent: an urgent procedure is one that is performed within 48 hours of upon diagnosis of the participant's status due to urgency of the medical condition, but is not otherwise considered emergent
- Emergent: an emergent procedure is one that is performed as soon as possible (usually within 6 hours)
- Elective: an elective procedure is one that is scheduled and is not urgent or emergent

Procedural Success may be defined as follows:

- CABG: the successful placement of at least one conduit with either a proximal and distal anastomosis or a distal anastomosis only
- PCI: successful balloon inflation with or without stenting and the achievement of a residual stenosis <30% as observed by QCA or <20% by visual estimation if QCA is not available. The balloon inflation and/or stenting could have been preceded by adjunctive device use (e.g., Angiojet, rotational atherectomy etc.)

Target Lesion (TL): A lesion revascularized in the index procedure (or staged procedure). The left-main target lesion extends from the distal left main stem to the end of the 5 mm proximal segments of the left anterior descending and left circumflex arteries as well as the ramus intermedius if the latter vessel has a vessel diameter of ≥2 mm.

Target Vessel (TV): The target vessel is defined as the entire major coronary vessel proximal and distal to the target lesion including upstream and downstream branches and the target lesion itself. The left main and any vessel originating from the left main coronary artery or its major branches is, by definition, considered a target vessel for the purposes of this trial (unless either the LAD or LCX are occluded at baseline and no attempt was made to Revascularized these territories by either PCI or CABG).

Target Vessel-Non-Target Lesion: The target vessel but non-target lesion consists of a lesion in the epicardial vessel/branch/graft that contains the target lesion; however, this lesion is outside of the target lesion by at least 5 mm distal or proximal to the target lesion determined by quantitative coronary angiography (QCA).

Non-Target Vessel: For the purposes of this trial, the only possible non-target vessel would be the right coronary artery and its major branches that were not treated by either PCI or CABG at the index procedure (unless either the LAD or LCX are occluded at baseline and no attempt was made to revascularize these territories by either PCI or CABG).

For Publication Purposes

Provided for Publication Purposes

Target Vessel Revascularization (TVR): Target vessel revascularization is any repeat percutaneous intervention of the target vessel or bypass surgery of the target vessel

Target Lesion Revascularization (TLR): Target lesion revascularization is defined as any repeat percutaneous intervention of the target lesion or bypass surgery of the target vessel

All TLRs will be recorded in the EDC and be classified prospectively as clinically-indicated or not clinically-indicated by the Investigator prior to the reintervention as well as retrospectively by the independent clinical event adjudication committee.

Clinically-Indicated (Repeat) Revascularization

A revascularization is considered clinically indicated if associated with any of the following:

- Positive invasive functional ischemia test (e.g. FFR, iFR, Doppler flow velocity reserve)
- Angiographic diameter stenosis ≥ 50% and positive non-invasive ischemia test (e.g. dobutamine stress test, nuclear test, exercise test, FFR-CT)
- Angiographic diameter stenosis ≥ 50% and ischemic symptoms (stable angina or acute coronary syndrome)
- Angiographic diameter stenosis ≥70%

When the diameter stenosis is <50%, the presence of severe ischemic signs and symptoms (e.g. acute myocardial infarction) would also confirm the diagnosis of a clinically-indicated revascularization.

Clinically indicated Revascularization Hierarchies for Assessment:

When invasive functional assessment is available, use the following hierarchy:

1. Core laboratory–reported fractional flow reserve ≤0.80 or instant wave-free ratio ≤0.89
2. Site-reported fractional flow reserve ≤0.80 or instant wave-free ratio ≤0.89

When percent diameter stenosis is available, use the following hierarchy:

1. Three-dimensional QCA analysis
2. QCA analysis based on the average of all available (preferably multiple) views
3. QCA analysis based on the worst view

For Publication Purposes

STENT THROMBOSIS per ARC-II definition [41]:

Classification	Criteria
Definite stent thrombosis	Angiographic confirmation of stent thrombosis*
	The presence of a thrombus† that originates in the stent/scaffold or in the segment 5 mm proximal or distal to the stent/scaffold or in a side branch originating from the stented/scaffolded segment and the presence of at least 1 of the following criteria:
	Acute onset of ischemic symptoms at rest
	New electrocardiographic changes suggestive of acute ischemia
	Typical rise and fall in cardiac biomarkers (refer to definition of spontaneous myocardial infarction)
	Or
	Pathological confirmation of stent thrombosis
	Evidence of recent thrombus within the stent determined at autopsy
	Examination of tissue retrieved following thrombectomy (visual/histology)
Probable stent thrombosis	Regardless of the time after the index procedure, any myocardial infarction that is related to documented acute ischemia in the territory of the implanted stent without angiographic confirmation of stent thrombosis and in the absence of any other obvious cause.‡
Silent stent occlusion	The incidental angiographic documentation of stent occlusion in the absence of clinical signs or symptoms is not considered stent thrombosis.
**Timing of Stent thrombosis (duration after stent implantation)**	
Acute	0h - 24 h
Subacute	>24 h – 30 days
Late	>30 days – 1 year
Very late	> 1 year

Early stent thrombosis is 0 to 30 days (acute plus subacute stent thrombosis).
MI indicates myocardial infarction.
**Definite stent/scaffold thrombosis is considered to have occurred by either angiographic or pathological confirmation.*
†Occlusive thrombus: Thrombolysis in Myocardial Infarction grade 0 or 1flow within or proximal to a stent/scaffold segment. Nonocclusive thrombus:intracoronary thrombus is defined as a (spherical, ovoid, or irregular) noncalcified filling defect or lucency surrounded by contrast material (on 3 sides or within a coronary stenosis) seen in multiple projections, persistence of contrast material within the lumen, or visible embolization of intraluminal material downstream.
‡When the stented/scaffolded segment is in the left circumflex coronary artery or in the presence of preexisting electrocardiographic abnormalities (eg, left bundle branch block, paced rhythms), definitive evidence of localization may be absent and Clinical Events Committee adjudication is based on review of all available evidence). Also, not that for this study, the SCAI definition is used for peri-procedural MI and the 4th UDMI for all other types.
§Defined as the moment the patient is undraped and taken off the catheterization table.

Provided for Publication Purposes

Investigator-reported HOSPITALIZATION for heart failure[43]

An event where the patient is admitted to the hospital with a primary diagnosis of HF where the length of stay is at least 24 h (or extends over a calendar date if the hospital admission and discharge times are unavailable), where the patient exhibits new or worsening symptoms of HF on presentation, has objective evidence of new or worsening HF, and receives initiation or intensification of treatment specifically for HF.
For time-dependent analysis the admission day is used as “date of HF”.

For Publication Purposes

Provided for Publication Purposes

OTHER DEFINITIONS

ANGINA per Braunwald classification [44,45] and Canadian Cardiovascular Society classification [46]

Braunwald classification of unstable angina
Severity

Class 1: New onset of severe or accelerated angina. Patients with new onset (< 2 months in duration) exertional angina pectoris that is severe or frequent (> 3 episodes/day) or patients with chronic stable angina who develop accelerated angina (that is, angina distinctly more frequent, severe, longer in duration, or precipitated by distinctly less exertion than previously) but who have not experienced pain at rest during the preceding 2 months.

Class 2: Angina at rest, subacute. Patients with one or more episodes of angina at rest during the preceding month but not within the preceding 48 hours.

Class 3: Angina at rest, acute. Patients with one or more episodes of angina at rest within the preceding 48 hours.

Clinical circumstances in which unstable angina occurs:

Class A: Secondary unstable angina. Patients in whom, unstable angina develops secondary to a clearly identified condition extrinsic to the coronary vascular bed that has intensified myocardial ischemia. Such conditions reduce myocardial oxygen supply or increase myocardial oxygen demand and include anaemia, fever, infection, hypotension, uncontrolled hypertension, tachyarrhythmia, unusual emotional stress, thyrotoxicosis, and hypoxemia secondary to respiratory failure.

Class B: Primary unstable angina. Patients who develop unstable angina pectoris in the absence of an extra-cardiac condition that have intensified ischemia, as in class A.

Class C: Post-infarction unstable angina. Patients who develop unstable angina within the first 2 weeks after a documented acute myocardial infarction.

For Publication Purposes

Provided for Publication Purposes

Canadian cardiovascular society classification of angina

Class 1: Ordinary physical activity does not cause angina, such as walking and climbing stairs. Angina with strenuous, rapid, or prolonged exertion at work or during recreation.

Class 2: Slight limitation of ordinary activity. Walking or climbing stairs rapidly, walking uphill, walking or stair climbing after meals, or in cold, or in wind, or under emotional stress, or any only during the first hours after awakening. Walking more than two blocks on the level and climbing more than one flight of ordinary stairs at a normal pace and in normal conditions.

Class 3: Marked limitations of ordinary physical activity. Walking one to two blocks on the level and climbing one flight of stairs in normal conditions and at a normal pace.

Class 4: Inability to carry on any physical activity without discomfort. Angina syndrome may be present at rest.

For Publication Purposes

DISSECTION, NHLBI CLASSIFICATION (National Heart Lung and Blood Institute)

Type A Small radiolucent area within the lumen of the vessel disappearing with the passage of the contrast material

Type B Appearance of contrast medium parallel to the lumen of the vessel disappearing within a few cardiac cycles

Type C Dissection protruding outside the lumen of the vessel persisting after passage of the contrast material

Type D Spiral shaped filling defect with or without delayed run-off of the contrast material in the antegrade flow

Type E Persistent luminal filling defect with delayed run-off of the contrast material in the distal lumen

Type F Filling defect accompanied by total coronary occlusion

For Publication Purposes
