# Supplementary material for: A multi-center, international, randomized, 2-year, parallel-group study to assess the superiority of IVUS-guided PCI versus qualitative angio-guided PCI in unprotected left main coronary artery (ULMCA) disease: Study protocol for OPTIMAL trial
Source: PLoS One. 2022 Jan 7;17(1):e0260770. doi: 10.1371/journal.pone.0260770 (PMC8740965; doi:10.1371/journal.pone.0260770)
Supplement: S1 File — (PDF) [file pone.0260770.s004.pdf]

The **OPTIMAL** Randomized Controlled Trial: OPtimizaTIon of Left MAin PCI with IntravascuLar Ultrasound.

Dear Sir/Madam,

We would like to invite you to participate in the above-mentioned clinical research study (from now on referred to as "the study"). It is important that you carefully consider your participation in the study, based on sufficient information. In this information sheet, all aspects of taking part in this study will be explained. Please take your time to read through this document and, if desired, take this document home and discuss the study with your family and/or friends. You are free to take as much as time as you need to decide about your participation. Should you, at any point, have more questions about the study or your participation, you are invited to contact your study doctor and/or the research team for more information. Their contact details are provided at the end of this information sheet.

The study has been reviewed and approved by an independent Ethics Committee.

Voluntary participation
It is up to you to decide whether or not to take part in this study. If you decide to take part, you will be given this information sheet to keep and be asked to sign the consent form. The original consent form will be stored at your hospital and you will receive a copy.
If you decide to take part, you are free to withdraw at any time and without giving a reason. This will not affect the standard of care you receive or the relation with your doctor. In case you would like to withdraw from the study, please inform your study doctor.
Please be aware that the study doctor, or your own cardiologist, can decide to end your participation in the study, without your prior approval, if this is deemed to be in your best interest.
If you withdraw from the study, your study doctor will no longer contact you for the study, but your study doctor will collect your vital status at the end of the study e.g. by contacting your general practitioner or accessing available registries. Collection of other data for study purposes will be stopped. However, all data collected before you withdraw, will remain part of the study. If you wish the deletion of your data, you can request to do so.

Introduction
Your doctor has explained to you that one or more of the blood vessels that supply the heart muscle with blood are narrowed or blocked. In the near future, whether you participate in this study or not, you will undergo a "percutaneous coronary artery intervention" (PCI). During this treatment, a stent (thin metal tube) will be inserted into the affected coronary artery via a catheter, which is inserted via the arm or groin. The stent is then used to push and keep the blood vessel open, so that blood flow is restored.
You recently underwent an angiography, a test that enables the doctors to look at your heart's blood supply vessels. During this angiography it was found that at least 1 of the obstructions is located in the main stem (a particular coronary artery), which makes you a potential candidate for the Optimal study.

Purpose of the Study
During the PCI your coronary arteries are visualized. These images help the cardiologist when placing the stent. There are multiple ways to create images of your coronary arteries. In this study 2 methods of visualizing your coronary arteries during the PCI are compared. The 2 methods compared in this study are:

<u>IVUS guided PCI:</u>
IVUS is an abbreviation for IntraVascular UltraSound. This is a procedure that uses echography (ultrasound) to visualize the inside of your blood vessels. The cardiologist performs this examination during the PCI (e.g.

For Publication Purposes

right before and/or after placing the stent). An echo-element is mounted on the tip of the catheter which is inserted via the arm or groin.

<u>Angiographic guided PCI:</u>
An angiography is a procedure that uses X-ray to visualize your blood vessels. The cardiologist performs this examination during the PCI (e.g. right before and/or after placing the stent). A contrast fluid is injected into the blood vessels via a catheter which is inserted via the arm or groin. At the same time, an X-ray image is made.

All patients will receive both the contrast dye, and the conventional angiography guided PCI. Those within the IVUS group will receive the procedure in addition to the angiography guided PCI.

Both methods are used in daily practice during PCI. This study aims to compare the IVUS guided PCI against the angiographic guided PCI for lesions located in the main stem coronary artery, and to see if one approach is better than the other.

Study Organization
This study is designed by investigators under the umbrella of the European Cardiovascular Research Institute (ECRI). ECRI is the sponsor of the study. Research grants were provided by Boston Scientific and Philips Volcano.

Approximately 800 patients will participate in this study. The study is conducted in approximately 25 hospitals in Italy, Spain and the United Kingdom.

Study Design
Because we do not know which method of visualizing the coronary arteries during PCI is the best, we need to make a comparison. For everyone who agrees to take part in this study, the visualization method will be chosen by a process called randomization. The method is randomly allocated by a computer, which is like making a choice by tossing a coin. This means that you have an equal chance of being allocated to one of the methods, either IVUS guided PCI or angiographic guided PCI.

You and your doctor will know which method for visualizing the coronary arteries is allocated to you. If, for any clinical reason, the cardiologist decides during the PCI that the other methods is better to be used, he is allowed to do so.

In this study only IVUS devices manufactured by Boston Scientific and Philips Volcano are used. The stent that is used to keep your coronary artery open is the Synergy system, manufactured by Boston Scientific.

Description of the Study
If you agree to participate in the study, the method used to visualize your blood vessels is defined by the randomization. The further preparation and execution of the PCI is done as per standard of care and not unique for this study. The examinations that will be performed and the medication that will be prescribed in preparation and directly after the PCI are also not unique for this study part. They are part of the standard of care for patients in your situation in your hospital. These examinations include blood sampling, an electrocardiogram (ECG), an echography of your heart and a physical examination.

In addition, the following phone contacts, hospital visits and examinations are expected to be done specifically because you participate in this study:

<u>1 month after your PCI: phone contact</u>
- You will be in contact by phone with your study doctor or another member of the research team at the hospital where the PCI was done.

For Publication Purposes

- During the phone call the following will be discussed:
    - How you are doing at that moment
    - The occurrence of any new diseases or new symptoms, since your PCI
    - Your medication intake

1 year and 2 years after your PCI: hospital visit or phone contact

- You will have an appointment with your study doctor and/or his research team in the hospital where the PCI was done, or you will be in contact by phone.
- During this appointment, the following will be discussed:
    - How you are doing at that moment
    - The occurrence of any new diseases or new symptoms, since your PCI
    - Your medication intake
- In case of a hospital visit, an electrocardiogram (ECG) will be made, which is a non-invasive test to record the electrical activity of your heart.

Additional phone contacts and/or hospital visits

- You are encouraged to contact your study doctor or the research team in case of any new diseases or new symptoms, also if these are treated by another doctor or in another hospital.
- It is possible that the sponsor considers it necessary to extend the period of follow up, up to a maximum of 5 years. If so, the sponsor will request approval of the independent ethics committee. You can then be requested for additional phone contacts and/or hospital visits. During these additional visits, the same points as described above will be discussed and examined.

What is expected from you?

If you decide to participate in the study, we would like to ask you to do the following:

- Follow the advice of your study doctor.
- After you have received the stent, you will be prescribed standard medication, consisting of Aspirin and 1 other medication, aimed to prevent the formation of blood clots. It is very important, for your own wellbeing, that you take the medication as instructed by your study doctor. Never stop taking the medication without receiving prior approval from your doctor.
- Perform the follow up visits and participate in the phone contacts as requested for the study and described above.

If deemed necessary for the study, the study doctor will contact your general practitioner or treating cardiologist for your medical records.

Benefits

Your participation may not benefit you directly, but the knowledge gathered as a result of this study is valuable to researchers and doctors for the treatment of future patients who will have a PCI. One advantage for you may be a closer monitoring of your medical condition.

Potential risks and discomforts

It is expected that the potential risks resulting from participation in this study are similar to the potential risks you would have when you receive the standard treatment for your condition. Your doctor can inform you in more detail about the potential risks associated with a PCI. There may be side effects that the researchers currently do not expect or do not know about.

In case of adverse events as a result from participating in the clinical study, additional health care will be offered.

New information

If any new information becomes available that might affect your willingness to participate, your doctor will inform you in writing. If relevant, you will be asked to confirm your continued consent.

For Publication Purposes

Alternative treatments
The PCI is a standard procedure for your condition. Both visualization methods (IVUS and angiography) are used with PCI in daily practice. Your doctor will inform you about other possible treatments. Should you decide not to participate in this study, you will likely still undergo PCI.

Confidentiality of your data
The study doctor will report relevant encoded data originating from your medical files to the company responsible for the processing of data used in this study. Copies of your angiogram and/or IVUS images (which may display your full name) may be sent to a central laboratory for analysis (Cardialysis in Rotterdam). Upon arrival at the central laboratory, your name and / or any identifying data will be removed, if present, and replaced by a number to guarantee your anonymity. These copied results will then be used for analysis by Cardialysis and forwarded to the main study investigators and the grant givers (Philips Volcano and Boston Scientific, whose main offices are located outside the EEA).

To verify study procedures and/or data, it might be necessary for representatives of the study sponsor, ethics committee, or regulatory authorities to have direct access to your medical records, including all identifying information, to the extent permitted by the applicable laws and regulations. If you do not agree, you cannot take part in this study.

How will we use information about you?
We will need to use information from you, your medical records and your GP for this research project.
This information will include: -

- Your Name/Initials
- NHS number
- Copies of your angiogram and/or IVUS images

People will use this information to do the research or to check your records to make sure that the research is being done properly.

People who do not need to know who you are will not be able to see your name or contact details. Your data will have a code number instead.
We will keep all information about you safe and secure.

Some of your information will be sent to Cardialysis in The Netherlands. They must follow our rules about keeping your information safe.

Once we have finished the study, we will keep some of the data so we can check the results. We will write our reports in a way that no-one can work out that you took part in the study.

What are your choices about how your information is used?

- You can stop being part of the study at any time, without giving a reason, but we will keep information about you that we already have.
- If you choose to stop taking part in the study, we would like to continue collecting information about your health from central NHS records, your hospital, your GP. If you do not want this to happen, tell us and we will stop.
- We need to manage your records in specific ways for the research to be reliable. This means that we won't be able to let you see or change the data we hold about you.

Your medical files will be kept in accordance with applicable legislation. All information pertaining to the study will be stored for 15 years after the end of the study, or shorter if 15 years is not allowed by local laws and regulation.

Where can you find out more about how your information is used?

For Publication Purposes

You can find out more about how we use your information:

- at www.hra.nhs.uk/information-about-patients/
- our leaflet available from www.hra.nhs.uk/patientdataandresearch
- by asking one of the research team
- by sending an email to the Data Protection Officer of Cardialysis at dpo@cardialysis.nl.

Cardialysis is, on behalf of the sponsor, responsible for the processing of your data.

Your General Practitioner and/or treating cardiologist will be informed about your participation in the study.

Termination of the study
The sponsor of the study (ECRI) or the Ethics Committee may decide to early terminate the study or your participation in the study, if they have significant reasons for that. In that case, you will be informed by your doctor. You will receive continued standard health care, the same as if you were not in the study.

Registration of the study
A description of this study is available on www.clinicaltrials.gov. This website does not contain any information that can identify you. A summary of the results will be placed on the website. You can find the study on this website under the number NCT04111770.

Are there any additional costs/is there any remuneration if you decide to take part in this study?
Your participation in the study will not entail any additional costs for you. You will not be paid for your participation.

Are you insured if you take part in the study?
The organisation responsible for the study, ECRI, does have specific insurance coverage for this study. In the unlikely event of an injury during your participation in this study as a result of the procedure, your doctor will treat you according to the local hospital practice. It is important, however, that you inform your doctor or the study research team of any change in your health or any other medical treatments that you may require during the course of this study. Your study doctor will then notify ECRI.
Since both methods of imagining during PCI are part of the standard of care, there is no additional insurance specific for the study.

Problems or questions
It is important that you understand all information about the study before you sign the form providing your voluntary consent to participate in the study.

If you have questions or complaints during the study, we ask that you contact the study doctor or your treating doctor.

The contact details are listed in Appendix 1.

Thank you for taking the time to read this document, it is recommended that you keep a copy of this document so that you may review it later if necessary.

For Publication Purposes

Appendix 1 : Contact information

Principal Investigator:

Dr Giovanni Luigi De Maria 01865 223 173

Research Staff:

Deborah Barker 01865 223 349 or Email: Deborah.Barker@ouh.nhs.uk

Bernadette Moreby 01865 223 349 or Email: Bernadette.Moreby@ouh.nhs.uk

For Publication Purposes

CONSENT FORM

Please initial all boxes

- I declare that I have been informed of the nature of the study, its purpose, its duration, any risks and benefits and what is expected of me. I have had sufficient time to consider my participation. I was able to ask questions and my questions have been answered. I have taken note of the information document and the appendices to this document. I can request additional information at any time. ☐

- I understand that my participation is voluntary and that I am free to withdraw at any time without giving any reason, and without my medical care or legal rights being affected. I understand that all data collected up until the moment of withdrawal will remain part of the study. ☐

- I understand that all documents that are part of my medical dossier will be handled with strict confidentiality. I also understand that my medical dossier may be reviewed by the representatives of the sponsor (ECRI), ethics committee or regulatory authorities. I give these persons permission to review my dossiers. ☐

- I consent to my GP and/or treating physician being informed about my participation in this study. ☐

- I consent to collection and processing of my data as described in the patient information and to my medical information pertaining to the study being stored for 15 years after the end of this study and being forwarded outside the EEA. ☐

- I consent to the transfer of images of my coronary arteries, collected just prior and during the study using different imaging techniques to Cardialysis (Rotterdam, the Netherlands) and to the main study investigators and grant givers (Philips Volcano and Boston Scientific). ☐

- I will receive a copy of the patient information and consent form signed by the doctor. ☐

- I voluntarily agree to take part in the Optimal study. ☐

☐

Patient

Name: ____

Signature: ______________________________Date: ___________ Time*______________________

For Publication Purposes

Study doctor or delegated person
I hereby declare that I have informed the patient about the goal, the potential risks and consequences of the Optimal study.

Name:____

Signature: _________________________________ Date: _________________________________

If a witness/interpreter is present

I was present during the entire process of informing the patient and I confirm that the information on the objectives and procedures of the study was adequately provided, that the participant apparently understood the study and that consent to participate in the study was freely given.
Name:

____

Signature: ________________________________Date: ___________ Time*_______________________

**Time is only required if you sign on the same day as the stent placement*

For Publication Purposes
